# Pathogenic POLRMT variants in mice impair mtDNA transcription and affect perinatal survival

**DOI:** 10.1172/jci.insight.199182

**Published:** 2026-07-22

**Authors:** David Alsina, Diana Rubalcava-Gracia, Kristina Bubb, Rodolfo Garcia-Villegas, Akos Vegvari, Roberta Filograna, Florian A. Rosenberger, Camilla Koolmeister, Nils-Göran Larsson

**Affiliations:** 1 Department of Medical Biochemistry and Biophysics, Karolinska Institutet, Stockholm, Sweden.; 2 Centre for Inherited Metabolic Diseases, Karolinska University Hospital, Stockholm, Sweden.; 3 Departamento de Biología Molecular y Biotecnología, Instituto de Investigaciones Biomédicas, Universidad Nacional Autónoma de México, Mexico City, Mexico.; 4 Department of Medical Biochemistry and Biophysics, Science for Life Laboratory, Karolinska Institutet, Stockholm, Sweden.

**Keywords:** Cell biology, Metabolism, Mitochondria, Mouse models

## Abstract

Mitochondrial gene expression is essential for oxidative phosphorylation that generates the bulk of the cellular ATP, and mitochondrial dysfunction is a common cause of human metabolic diseases. Recently, the first pathogenic variants in the only known mitochondrial RNA polymerase (POLRMT) were described in patients presenting with a wide variety of clinical manifestations, including hypotonia, short stature, and developmental delay. Here, we modeled two human pathogenic POLRMT variants by creating the corresponding substitutions in mice: the dominant S582F and the recessive R984C variant. Mice homozygous for the R984C variant showed perinatal lethality without apparent embryonic developmental defects, a finding consistent with a failure to adapt to the metabolic transition to oxidative metabolism at birth. Mice carrying the S582F variant were viable and exhibited decreased mitochondrial transcript levels due to impaired de novo transcription. However, mtDNA levels and *in organello* mtDNA replication remained normal, which recapitulates the molecular phenotypes observed in patients. Altogether, our findings indicate that the conserved arginine near the active site is essential for POLRMT function, while the serine in the intercalating hairpin of the N-terminal domain is required for near-genome length transcription but not primase activity. This study highlights genotype-phenotype differences and provides new insights into POLRMT function.

## Abstract 2

We describe two novel mouse models for inherited mitochonrial disease where the expresion of mtDNA is deficient.

## Introduction

Mitochondria are vital for energy conversion and metabolism, and their own genome (mtDNA) encodes 13 proteins that are essential core components of the mammalian oxidative phosphorylation (OXPHOS) complexes (1). Thus, defects in mtDNA-encoded proteins directly impact ATP production, and without proper mtDNA expression the system collapses (2, 3). In humans, mitochondrial malfunction causes metabolic disease and is closely linked to premature aging processes (4, 5). Most of the approximately 1,200 mitochondrial proteins are encoded by the nuclear genome and imported into mitochondria, including all proteins required for transcribing and replicating mtDNA (6, 7). The only known mitochondrial RNA polymerase (POLRMT) is a single-subunit enzyme required for both mitochondrial gene expression and priming of mtDNA replication (8, 9).

Mitochondrial transcription begins when the mitochondrial transcription factor A (TFAM) recruits POLRMT and the mitochondrial transcription factor B2 (TFB2M) to form a tripartite transcription initiation complex (1). After initiation, TFAM and TFB2M leave the complex and POLRMT transitions to elongation by associating with the mitochondrial transcription elongation factor (TEFM) (10, 11). Thus, the mitochondrial transcription machinery is distinct from bacterial and mammalian nuclear systems, and POLRMT plays an essential role in both mtDNA gene expression and replication (12). In mice, a full-body POLRMT knockout results in mid-gestation embryonic lethality, and a heart-specific knockout causes severe mitochondrial dysfunction with dilated cardiomyopathy and reduced mitochondrial mRNA and mtDNA levels (9).

POLRMT is related to the T7 bacteriophage polymerase but presents unique features. It requires additional proteins to recognize the promoters and maintains its initial conformation when it proceeds into elongation (1). Furthermore, POLRMT contains a non-conserved N-terminal extension (NTE) followed by a pentatricopeptide repeat (PPR) domain of unknown function. The NTE functions as an inhibitory domain and interacts with TFAM for initiation of promoter-specific transcription (13). Similarly to the T7 RNA polymerase, POLRMT contains a conserved N-terminal domain (NTD) and a conserved C-terminal domain (CTD). The NTD contains an intercalating hairpin that contacts TFB2M and separates the mtDNA strands at the active center cleft of POLRMT. The CTD contains the catalytic core of the enzyme consisting of the palm, fingers, and thumb regions (14). Transcription initiation has been reported to be regulated through a negative feedback loop involving POLRMT dimerization induced by 7S RNA. The promoter-proximal 7S RNA is transcribed from the mtDNA light strand promoter and is polyadenylated and can therefore not hybridize with mtDNA. To activate POLRMT, the mtEXO complex degrades the 7S RNA, thereby relieving the inhibition and allowing transcription to resume (15).

Until recently, no mitochondrial disease-causing variants had been reported in the *POLRMT* gene, which is in sharp contrast to the hundreds of pathogenic variants of the mitochondrial DNA polymerase (*POLG*) gene (16). Recent studies reported several mitochondrial disease patients from unrelated families with pathogenic variants in *POLRMT* (17, 18). Clinical manifestations vary widely, but developmental delay, hypotonia, short stature, and intellectual disability are the most common findings. Most of the described patients carry compound heterozygous variants. These variants are located in the PPR, the NTD, and the CTD domains, including residues near the active site (17, 18). The *POLRMT* mutations can be either recessive or dominant, and molecular analyses of several of the corresponding mutant POLRMT proteins have been performed (17). Mitochondrial transcript levels are reduced to variable degrees whereas mtDNA copy number abnormalities are rare in affected patients. The normal mtDNA levels in patients with *POLRMT* mutations likely explain why the clinical manifestations are different from those of patients with *POLG* mutations, who typically have mtDNA depletion or deletions, causing, e.g., Alpers syndrome, epilepsy, liver failure, ataxia, and chronic progressive external ophthalmoplegia (19).

Here, we investigated the impact of two pathogenic human POLRMT variants by using CRISPR/Cas9 genome editing to generate the corresponding mutations in mice. We selected these mutations based on their localization within functional sites. The dominant S611F variant affects the intercalating hairpin, and the recessive R1013C variant is located in close vicinity to the active site of POLRMT. Both variants showed highly reduced in vitro activity (17). We performed in-depth characterizations of these mouse models at both the gross and molecular levels to explore how alterations in the transcriptase and primase functions of POLRMT influence development and adult physiology. This work provides valuable insight into the mechanistic roles of POLRMT in mitochondrial gene expression and advances our understanding of the molecular basis of human diseases caused by these mutations.

## Results

### Generation of mouse lines carrying pathogenic POLRMT variants.

**Figure 1:**
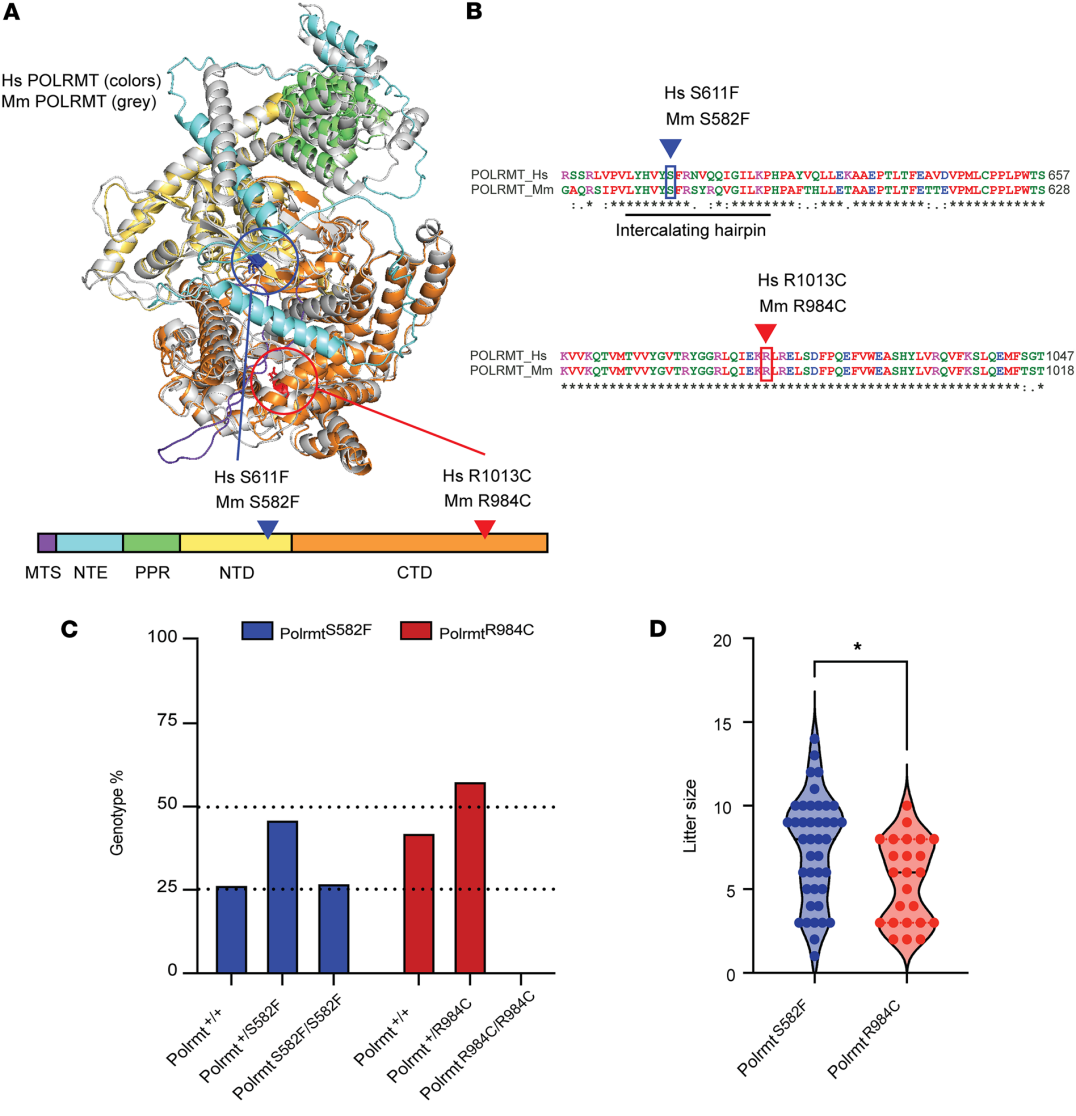
Modeling pathogenic POLRMT variants in mice. (**A**) The cryogenic electron microscopic (cryo-EM) structure of human (Hs) POLRMT (colors; Protein Data Bank ID: 6ERQ) was superimposed to the structure of mouse (Mm) POLRMT generated by AlphaFold (gray). The domains of human POLRMT are color-coded as indicated. The location of the POLRMT pathogenic variants, and the corresponding residues in mouse, are indicated by blue (S611F) and red (R1013C) circles and arrowheads. MTS, mitochondrial targeting sequence; NTE, N-terminal extension; PPR, pentatricopeptide repeat domain; NTD, N-terminal domain; CTD, C-terminal domain. (**B**) Partial amino acid sequence alignment of human (Hs) and mouse (Mm) POLRMT, highlighting the positions of the substituted residues. The locations of the S611F and R1013C variants, and their corresponding mouse residues (S582F and R984C), are boxed and indicated by arrowheads. Asterisks denote identical residues, colons indicate conserved residues, and periods represent semi-conserved residues. Alignment was performed with Clustal Omega (https://www.ebi.ac.uk/jdispatcher/msa/clustalo). (**C**) Bar graph showing the genotypic distribution of offspring from the *Polrmt*-knockin mouse lines carrying the S582F (blue) and R984C (red) mutations at weaning. The observed proportions of each genotype are plotted. Dotted lines represent the expected Mendelian ratios: 25% wild type, 50% heterozygous, and 25% homozygous knockin. S582F, *n* = 305 pups; R984C, *n* = 132 pups. (**D**) Violin plot showing the distribution of litter sizes from *Polrmt*-knockin mouse lines carrying the S582F (blue) and R984C (red) mutations. *Statistically significant difference in litter size (Mann-Whitney test *P* value = 0.0101). Sample size: S582F, *n* = 41 litters; R984C, *n* = 24 litters.

We aimed to model the first reported patient variants of POLRMT causing mitochondrial disease (17). To this end, we generated 2 mouse lines by CRISPR/Cas9 genome editing, each carrying a pathogenic variant in POLRMT. The dominant human variant S611F corresponds to residue S582F in mouse POLRMT; the affected serine is in the conserved intercalating hairpin located in the NTD of POLRMT (Figure 1, A and B). The recessive human R1013C variant corresponds to residue R984C in mouse POLRMT and affects a conserved arginine in close vicinity to the active site located in the CTD (Figure 1, A and B) (14). Both knockin mutations were successfully introduced to mice by CRISPR/Cas9 editing of embryos, and we obtained viable homozygous S582F knockin mice (*Polrmt^S582F/S582F^*) but no homozygous R984C mice (*Polrmt^R984C/R984C^*) (Figure 1C). Accordingly, the litter sizes were significantly smaller when the heterozygous *Polrmt^R984C^* line was intercrossed in comparison with when the heterozygous *Polrmt^S582F^* line was intercrossed (Figure 1D), showing that the homozygous S582F variant is well tolerated in mice, whereas the homozygous R984C variant is lethal.

### Perinatal lethality of homozygous R984C mice.

**Figure 2:**
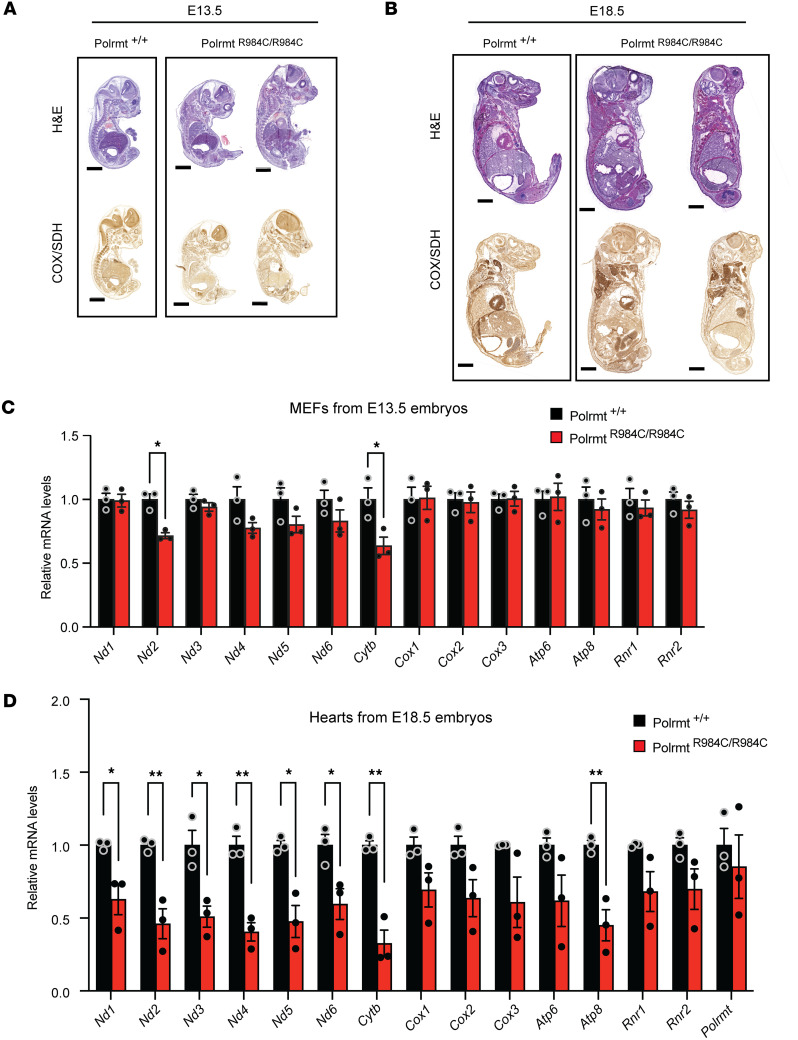
Cryosections and stainings of mouse embryos from the *Polrmt^R984C^* line. (**A**) Representative images of mouse embryos from the *Polrmt^R984C^* line dissected at embryonic day 13.5 (E13.5). Embryos were cryosectioned along the mid-sagittal plane and stained with hematoxylin and eosin (H&E; top) to assess overall morphology, or for cytochrome *c* oxidase (COX) and succinate dehydrogenase (SDH) activity (bottom) to evaluate mitochondrial function. *N* = 5–6 biological replicates per genotype. Scale bars: 2 mm. (**B**) Representative images of mouse embryos from the *Polrmt^R984C^* line dissected at E18.5. Embryos were cryosectioned along the mid-sagittal plane and stained with H&E (top) to assess overall morphology, or for COX and SDH activity (bottom) to evaluate mitochondrial function. *N* = 2 biological replicates per genotype. Scale bars: 2 mm. (**C**) RT-qPCR analysis of mitochondrial RNA transcripts in primary mouse embryonic fibroblasts (MEFs) derived from wild-type (+/+) and homozygous (R984C/R984C) E13.5 embryos. Relative expression levels were normalized to *Actb*. Data are presented as mean ± SEM. Statistical significance was determined using multiple *t* tests; *P < 0.05. *n* = 3 biological replicates per genotype. (**D**) RT-qPCR analysis of mitochondrial RNA transcripts in hearts from wild-type (+/+) and homozygous (R984C/R984C) E18.5 embryos. Relative expression levels were normalized to *Actb*. Data are presented as mean ± SEM. Statistical significance was determined using multiple *t* tests; *P < 0.05, **P < 0.01. *n* = 3 biological replicates per genotype.

To identify any defects during the embryonic development of the *Polrmt^R984C/R984C^* mice, we dissected staged embryos at embryonic day (E) 13.5 and E18.5 and observed no obvious pathology. Genotyping of 3 litters at E18.5 (*n* = 32 embryos) revealed a genotype distribution close to the expected Mendelian ratios (*Polrmt^+/+^* = 25%, *Polrmt^+/R984C^* = 46.9%, and *Polrmt^R984C/R984C^* = 28.1%). Cryosectioning of embryos followed by hematoxylin and eosin staining did not reveal any marked developmental defects at either of the two investigated stages (Figure 2, A and B). Cytochrome *c* oxidase (COX) and succinate dehydrogenase (SDH) double staining (COX/SDH) of E13.5 and E18.5 embryos did not show any blue cells or the characteristic mosaic pattern of tissues harboring mitochondrial dysfunction due to defective COX activity (Figure 2, A and B). Even though we observed expected Mendelian ratios and a lack of obvious defects during late embryonic development (E18.5), no homozygous *Polrmt^R984C/R984C^* pups were present at the time of weaning (P21). Given that no dead or sick *Polrmt^R984C/R984C^* neonates were recovered during routine cage checks, the likely explanation is that mutant pups die shortly after birth and are subject to maternal cannibalism (20, 21). Thus, while embryonic development is apparently normal, the time point of lethality likely occurs in the postnatal period and may be explained by poor ability to adapt to oxidative metabolism.

To further characterize the onset of the transcriptional defect, we analyzed mitochondrial transcript levels at different developmental stages. In primary mouse embryonic fibroblasts (MEFs) derived from E13.5 *Polrmt^R984C/R984C^* embryos, most mitochondrial transcripts remained largely unchanged in comparison with controls (Figure 2C). In contrast, we found a significant decrease of about 30%–60% in mtDNA transcript levels in the hearts of *Polrmt^R984C/R984C^* embryos compared with wild-type littermates at E18.5 (Figure 2D).

These findings suggest that the R984C mutation results in a progressive transcriptional deficiency. To test whether these mutant cells are specifically sensitive to increased metabolic demand, we cultured *Polrmt^R984C/R984C^* MEFs in galactose-supplemented, glucose-free medium to induce a shift toward oxidative metabolism. Mutant MEFs exhibited reduced proliferation under both standard and oxidative conditions (Supplemental Figure 1A; supplemental material available online with this article; https://doi.org/10.1172/jci.insight.199182DS1), indicating a latent sensitivity that likely becomes fatal during the physiological transition to oxidative metabolism at birth.

Taken together, these results show that *Polrmt^R984C/R984C^* embryos maintain sufficient transcriptional activity to allow apparently normal embryonic development, but these transcript levels are insufficient to support the metabolic shift to the oxidative metabolism that occurs postnatally.

### Mice harboring the S582F POLRMT variant have preserved motor performance.

**Figure 3:**
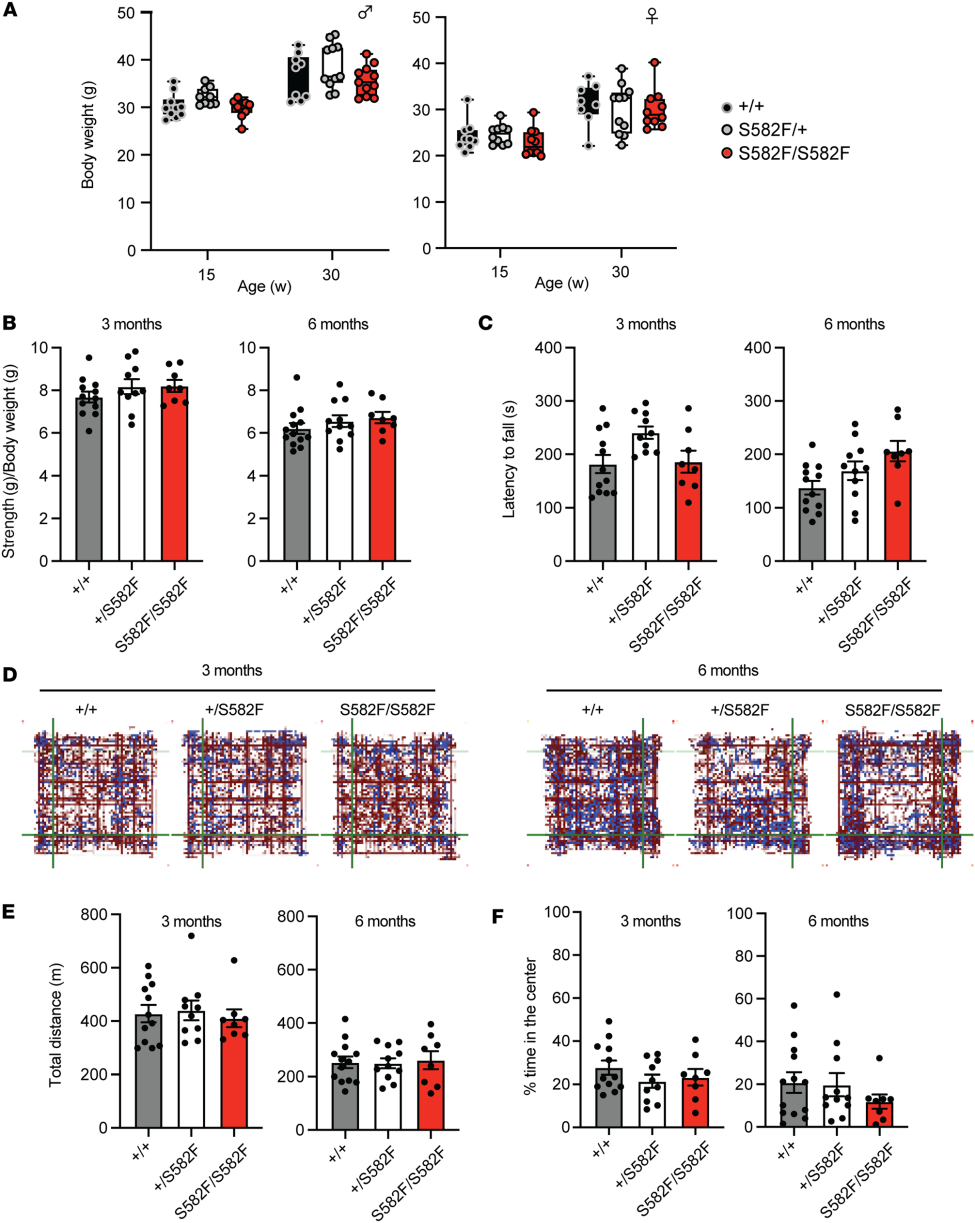
Phenotypic characterization of *Polrmt^S582F^* mice. (**A**) Body weights of male and female mice at 15 and 30 weeks of age. Data are presented as box-and-whisker plots. Boxes represent the interquartile range (25th to 75th percentiles) with the median value. Whiskers represent minimal and maximal values. *n* = 8–11 biological replicates per group. (**B**) Grip tests in wild-type (+/+), heterozygous (+/S582F), and homozygous (S582F/S582F) *Polrmt*-knockin male mice at 3 and 6 months of age. Data are represented as mean ± SEM. Data points represent biological replicates (3 months: +/+, *n* = 12; +/S582F, *n* = 10; S582F/S582F, *n* = 8; 6 months: +/+, *n* = 13; +/S582F, *n* = 11; S582F/S582F, *n* = 8). (**C**) Rotarod tests in wild-type (+/+), heterozygous (+/S582F), and homozygous (S582F/S582F) *Polrmt*-knockin male mice at 3 and 6 months of age. Data are represented as mean ± SEM. Data points represent biological replicates (3 months: +/+, *n* = 12; +/S582F, *n* = 10; S582F/S582F, *n* = 8; 6 months: +/+, *n* = 12; +/S582F, *n* = 11; S582F/S582F, *n* = 8). (**D**) Representative image of open field tracks. One image for each genotype and time point is shown. (**E**) Open field tests. Total distance covered over 60 minutes in wild-type (+/+), heterozygous (+/S582F), and homozygous (S582F/S582F) *Polrmt*-knockin male mice at 3 and 6 months of age. Data are represented as mean ± SEM. Data points represent biological replicates (3 months: +/+, *n* = 12; +/S582F, *n* = 10; S582F/S582F, *n* = 8; 6 months: +/+, *n* = 13; +/S582F, *n* = 11; S582F/S582F, *n* = 8). (**F**) Open field tests. Percentage of time spent at the center of the arena after 60 minutes in wild-type (+/+), heterozygous (+/S582F), and homozygous (S582F/S582F) *Polrmt*-knockin male mice at 3 and 6 months of age. Data are represented as mean ± SEM. Data points represent biological replicates (3 months: +/+, *n* = 12; +/S582F, *n* = 10; S582F/S582F, *n* = 8; 6 months: +/+, *n* = 13; +/S582F, *n* = 11; S582F/S582F, *n* = 8).

We characterized overall physiology and performance in the viable heterozygous (*Polrmt^+/S582F^*) and homozygous (*Polrmt^S582F/S582F^*) knockin mice harboring the POLRMT S582F mutation. Male and female knockin mice gained weight at comparable rates to wild types when analyzed at 15 and 30 weeks of age (Figure 3A). We performed a battery of behavioral experiments to assess neuromuscular function using grip strength tests (Figure 3B), motor coordination using rotarod tests (Figure 3C), and spontaneous locomotor activity and behavior using open field tests (Figure 3, D–F). We found that all of these parameters were comparable in *Polrmt^+/S582F^*, *Polrmt^S582F/S582F^*, and wild-type mice at 3 and 6 months of age.

### The S582F variant in mouse POLRMT recapitulates patient molecular phenotypes.

**Figure 4:**
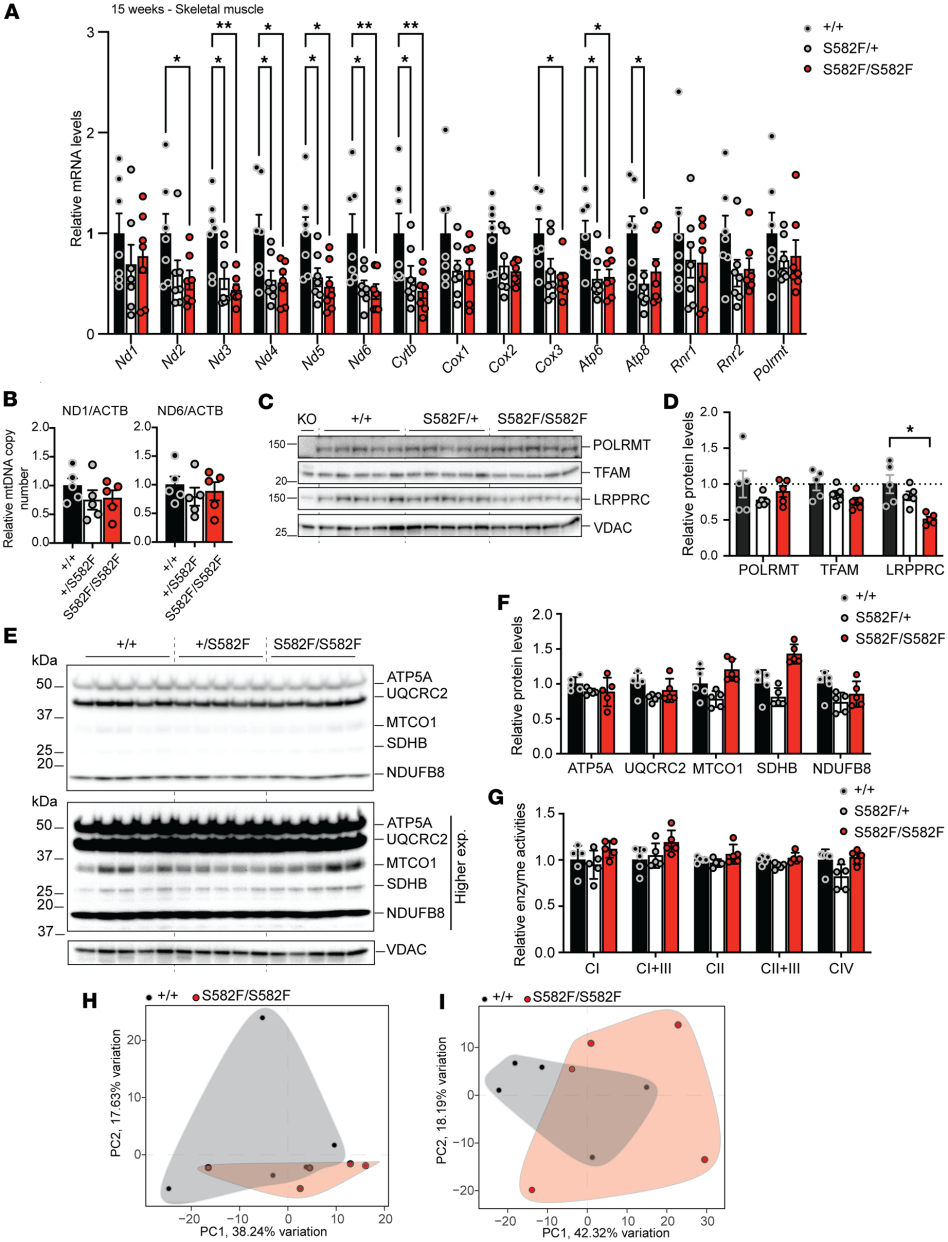
Molecular characterization of *Polrmt^S582F^* mice. (**A**) Relative transcript levels in skeletal muscle from wild-type (+/+), heterozygous (+/S582F), and homozygous (S582F/S582F) *Polrmt*-knockin mice at 15 weeks of age. Data are presented as mean ± SEM, *n* = 7 biological replicates per group. Two-way ANOVA followed by Dunnett’s multiple-comparison test, *P < 0.05, **P < 0.01. (**B**) Relative mtDNA copy number in skeletal muscle from wild-type (+/+), heterozygous (+/S582F), and homozygous (S582F/S582F) *Polrmt*-knockin mice at 15 weeks of age. Data are presented as mean ± SEM, *n* = 5 biological replicates. (**C**) POLRMT, TFAM, and LRPPRC protein levels in skeletal muscle mitochondria from wild-type (+/+), heterozygous (+/S582F), and homozygous (S582F/S582F) *Polrmt*-knockin mice at 15 weeks of age. A *Polrmt*-knockout (KO) sample was included as a negative control. *N* = 5 biological replicates per genotype. (**D**) Densitometric quantification of the Western blots in **C**. Data are plotted as fold change relative to wild-type samples and presented as mean ± SEM, *n* = 5 biological replicates per group. Two-tailed Student’s *t* test, *P < 0.05. (**E**) Analysis of OXPHOS subunit protein levels in skeletal muscle mitochondria from wild-type (+/+), heterozygous (+/S582F), and homozygous (S582F/S582F) *Polrmt*-knockin mice at 15 weeks of age. A high exposure of the same blot is shown in the lower panel. *N* = 5 biological replicates per genotype. (**F**) Densitometric quantification of the Western blots in **E**. Data are plotted as fold change relative to wild-type samples and presented as mean ± SEM, *n* = 5 biological replicates per group. (**G**) Respiratory chain complex enzymatic activities in skeletal muscle mitochondria from wild-type (+/+), heterozygous (+/S582F), and homozygous (S582F/S582F) *Polrmt*-knockin mice at 15 weeks of age. *N* = 5 biological replicates. (**H** and **I**) Principal component analysis (PCA) of the total proteome from heart (**H**) and liver (**I**) tissues of 15-week-old wild-type (black/gray) and *Polrmt^S582F/S582F^* (red) mice (*n* = 5 per group).

Next, we characterized the molecular phenotypes of *Polrmt^+/S582F^* and *Polrmt^S582F/S582F^* mice, and found a global reduction in mt-mRNA levels by RT-qPCR in skeletal muscle (Figure 4A). This decrease is in line with the dominant effect of the corresponding S611F mutation in human patients. Despite the global mt-RNA reduction in skeletal muscle, mtDNA levels were maintained, and the levels of TFAM protein, which binds and stabilizes mtDNA to form mitochondrial nucleoids (22), remained unchanged (Figure 4, B–D). Notably, the levels of the mt-mRNA–stabilizing protein LRPPRC were reduced (Figure 4, C and D), likely because of the decrease of the mt-mRNA levels. The steady-state levels of the mutant POLRMT protein were unchanged (Figure 4, C and D), showing that the S582F variant does not affect the stability of POLRMT, which is in contrast to many pathogenic POLG mutations that typically impair protein stability (23). To assess whether the reduced mitochondrial transcript levels affected the OXPHOS complexes, we measured the steady-state levels of protein subunits by Western blotting in isolated mitochondria. We did not observe any apparent decrease in OXPHOS protein levels (Figure 4, E and F), and consistently, the enzyme activities of the different respiratory chain complexes measured by spectrophotometry remained unaltered (Figure 4G).

### Proteomic analysis reveals robust mitochondrial proteostasis.

To investigate whether the reduction of mtDNA-encoded transcripts triggered tissue-specific proteomic remodeling, we performed label-free, quantitative proteomics on heart and liver tissues. In both heart (Figure 4H) and liver (Figure 4I), principal component analysis (PCA) showed substantial overlap between genotypes, with no clear separation along PC1 or PC2. Consistent with these findings, bulk proteomics revealed no significant differentially abundant proteins, indicating that the S582F mutation does not induce global proteomic remodeling in these tissues.

**Figure 5:**
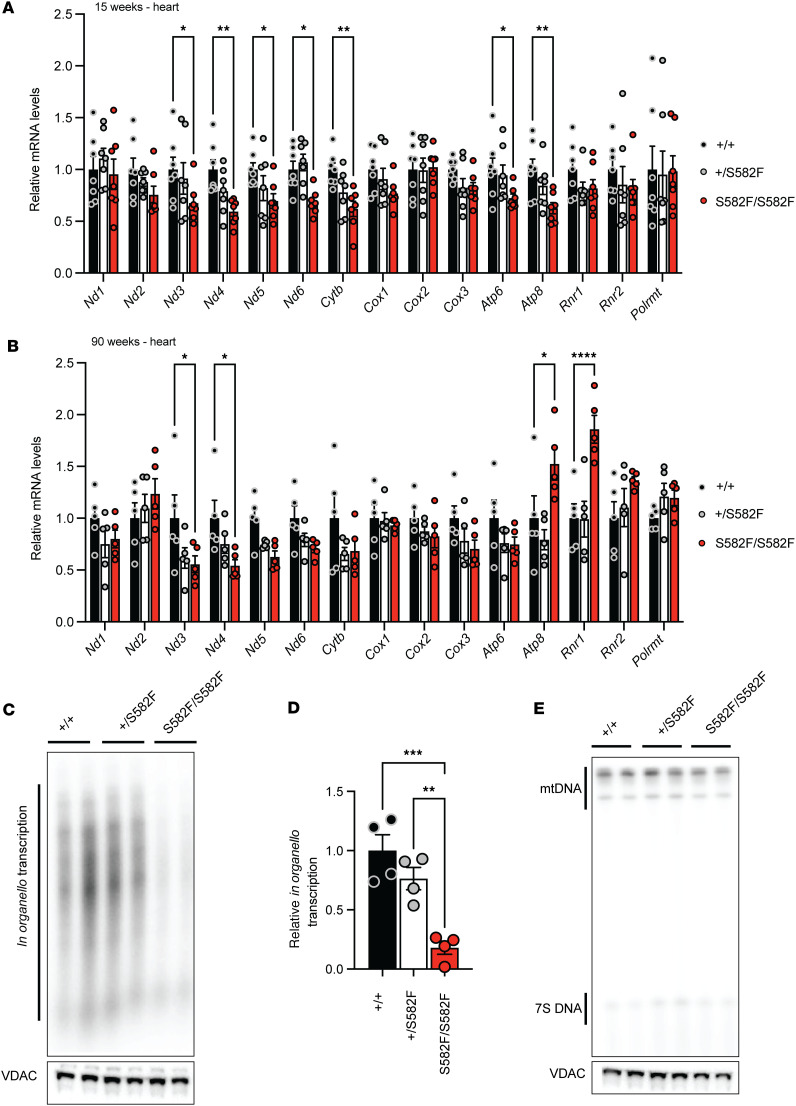
Age-dependent decline in mitochondrial transcript levels and *in organello* transcription activity in *Polrmt*-knockin mice. (**A**) Mitochondrial and *Polrmt* transcript levels in heart from wild-type (+/+), heterozygous (+/S582F), and homozygous (S582F/S582F) *Polrmt*-knockin mice at 15 weeks of age. Transcript levels were measured by RT-qPCR, normalized to *Actb*, and plotted relative to the wild-type group. Data are presented as mean ± SEM, *n* = 7 biological replicates per group. Data were analyzed with 2-way ANOVA followed by Dunnett’s multiple-comparison test, *P <0.05, **P < 0.01. (**B**) Mitochondrial and *Polrmt* transcript levels in heart from wild-type (+/+), heterozygous (+/S582F), and homozygous (S582F/S582F) *Polrmt*-knockin mice at 90 weeks of age. Transcript levels were measured by RT-qPCR, normalized to *Actb*, and plotted relative to the wild-type group. Data are presented as mean ± SEM, *n* = 5 biological replicates per group. Data were analyzed with 2-way ANOVA followed by Dunnett’s multiple-comparison test, *P < 0.05, ****P < 0.0001. (**C**) *In organello* synthesized mitochondrial transcripts were radiolabeled in isolated heart mitochondria from wild-type (+/+), heterozygous (+/S582F), and homozygous (S582F/S582F) *Polrmt*-knockin mice at 90 weeks of age. Input was monitored by Western blotting for voltage-dependent anion channel (VDAC) after labeling. A representative experiment is shown. (**D**) Densitometric quantification of *in organello* transcription activity. Signal from every lane was normalized to its respective VDAC signal and plotted relative to the wild-type group. Data are presented as mean ± SEM, *n* = 4 biological replicates per group. Data were analyzed with 1-way ANOVA followed by Dunnett’s multiple-comparison test, **P < 0.01, ***P < 0.001. (**E**) De novo synthesized DNA was radiolabeled in isolated heart mitochondria from wild-type (+/+), heterozygous (+/S582F), and homozygous (S582F/S582F) *Polrmt*-knockin mice at 90 weeks of age. Input was monitored by Western blotting for VDAC after labeling.

Moreover, targeted analysis of all OXPHOS subunits and key regulators of mtDNA expression, including TFAM, TFB2M, TEFM, LRPPRC, and SLIRP, revealed no significant differences in steady-state levels in these tissues (Supplemental Figure 2, A–D). These findings show that while transcript levels are reduced in skeletal muscle (Figure 4A) and more moderately reduced in the heart (Figure 5A), the proteome remains stable across tissues. This suggests a robust physiological buffering system whereby mice maintain mitochondrial proteostasis despite reduced mRNA availability, explaining the lack of an overt pathological phenotype.

### The S582F molecular phenotype does not progress in aged mice.

To assess whether the observed phenotype worsens with age, we analyzed a group of animals at 90 weeks of age. We measured mitochondrial transcript levels in different organs and found decreased mt-RNA levels in the heart (Figure 5, A and B), while the liver showed only a mild reduction or no reduction (Supplemental Figure 3, A and B), demonstrating that the pathogenic *Polrmt^S582F^* variant has tissue-specific effects. Although there was no clear age-related decline in mt-RNA levels in *Polrmt^+/S582F^* and *Polrmt^S582F/S582F^* mice, we observed higher variability, especially in skeletal muscle of aged animals (Supplemental Figure 3C). Interestingly, we noticed that some transcripts were stabilized rather than further decreased and even were present at higher levels in heart of *Polrmt^+/S582F^* and *Polrmt^S582F/S582F^* mice in comparison with wild types (Figure 5, A and B). This increase of steady-state transcript levels was especially noticeable for the ribosomal RNAs (*Rnr1* and *Rnr2*) in both heart and skeletal muscle.

We assessed the de novo mtDNA transcription and replication in isolated mitochondria from hearts of 90-week-old *Polrmt^+/S582F^* and *Polrmt^S582F/S582F^* mice. We observed drastically reduced de novo transcription in *Polrmt^S582F/S582F^* mitochondria, while *Polrmt^+/S582F^* mitochondria showed no significant reduction in comparison with wild-type mitochondria (Figure 5, C and D). In contrast, mtDNA de novo replication assays did not show any defects in *Polrmt^+/S582F^* and *Polrmt^S582F/S582F^* mitochondria in comparison with controls (Figure 5E). These findings are consistent with previous reports that low levels of POLRMT are able to support mtDNA replication in the heart of the mouse although gene expression is severely inhibited (9).

### The S582F mutation compromises cellular proliferation.

To further probe for latent defects that might only emerge under a stress challenge, we performed metabolic stress tests using primary MEFs derived from *Polrmt^S582F/S582F^* embryos.

**Figure 6:**
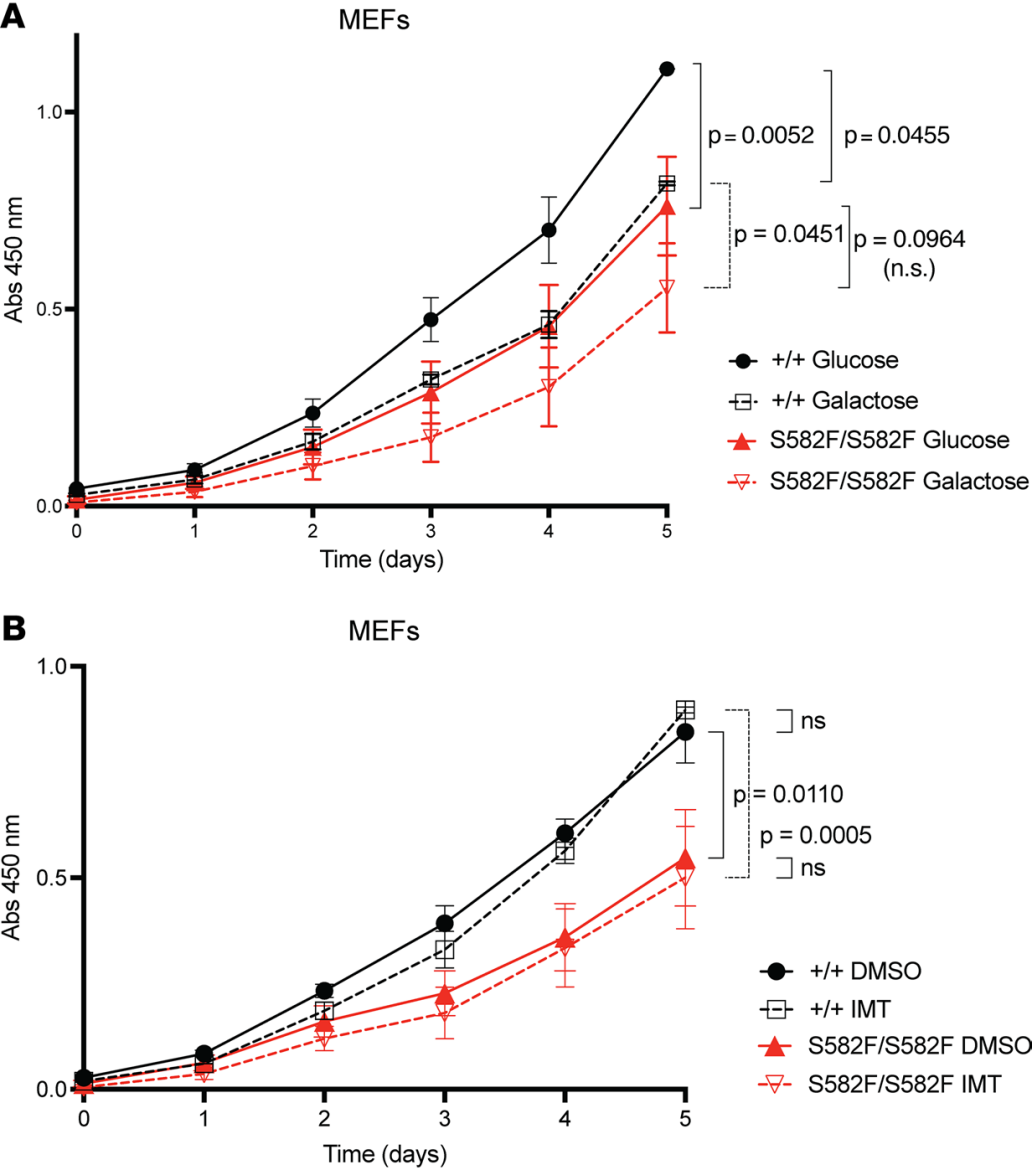
The *Polrmt* S582F mutation compromises cellular proliferation in MEFs. (**A**) Proliferation of wild-type (+/+) and homozygous (S582F/S582F) primary MEFs cultured in either glucose-containing (solid lines) or galactose-containing (dashed lines) medium over 5 days. (**B**) Proliferation of wild-type and mutant MEFs treated with either DMSO (control) or 100 nM of the POLRMT-specific inhibitor IMT1B (IMT). In both panels, cell viability and proliferation were measured using a CCK-8 kit. Data are presented as mean ± SEM. Statistical significance was determined by 2-way ANOVA with Tukey’s multiple-comparison test; *P* values for the day 5 time point are indicated on the graphs. *n* = 2–3 biological replicates per condition.

Under both glucose-containing and galactose-containing culture conditions, mutant MEFs exhibited impaired proliferation compared with wild-type controls (Figure 6A). When galactose is substituted for glucose, cells are forced to rely on OXPHOS for ATP production. The attenuation of growth in both conditions indicates that the S582F variant becomes limiting under proliferative conditions and under a high metabolic demand.

To assess whether the system could be further sensitized, we challenged the MEFs with the POLRMT-specific inhibitor IMT1B. We asked whether further pharmacological suppression of mitochondrial transcription acts synergistically with the S582F mutation to compromise cell proliferation. Interestingly, we observed no additional growth impairment upon treatment with IMT1B (Figure 6B and Supplemental Figure 4, A–C). The lack of an additive effect suggests that under these culture conditions, cells can rely on glycolysis for proliferation, and that suppression of POLRMT activity does not translate into a further growth defect.

## Discussion

Replication and transcription of mammalian mtDNA depend on POLG and POLRMT, respectively, both essential mitochondrial polymerases that have no known roles in the nucleus (9, 24, 25). While over 300 pathogenic *POLG* mutations (https://portal.niehs.nih.gov/polg/) cause mtDNA maintenance issues, leading to a wide variety of clinical phenotypes (19, 26), pathogenic *POLRMT* and *TEFM* variants were only recently identified (17, 27). A key finding in our study is that although POLRMT is required to generate the RNA primers to start mtDNA replication (1, 9), mtDNA copy number was preserved in *Polrmt^S582F/S582F^* mice, as well as in patient fibroblasts carrying *POLRMT* variants (17). These findings are consistent with experimental studies in the mouse showing that RNA primer formation for mtDNA replication can be maintained even at low POLRMT levels (9) but mice completely lacking POLRMT exhibit mtDNA depletion (9). This suggests that even a low residual polymerase activity is sufficient to sustain primer synthesis, explaining why *POLRMT* clinical phenotypes differ so significantly from *POLG* and other mtDNA depletion syndromes (19, 28).

Our *Polrmt^R984C/R984C^* model points to a critical vulnerability at birth. These embryos develop normally despite a mutation near the catalytic core of POLRMT but cannot survive the neonatal window. Studies on mouse models indicate two stages when mitochondrial function is critical for viability: first, when the maternal mtDNA contribution is diluted, and second, at birth when metabolism shifts toward respiration. Our findings are consistent with those reported in other mitochondrial deficiency models, such as mtDNA mutator or *Fbxl4*-knockout mice (29, 30), where lethality manifests at the neonatal window. Although the exact time point of lethality could not be narrowed down to a specific perinatal hour, our data indicate that lethality occurs in the early postnatal period. Our results show that while E13.5 MEFs maintain relatively stable mitochondrial transcripts, E18.5 hearts show a marked defect. This suggests that the R984C mutation causes a progressive transcriptional insufficiency that only becomes limiting upon the metabolic shift to oxidative metabolism at birth. In vitro transcription assays of the corresponding human variant (POLRMT^R1013C^) showed severely reduced activity (17), supporting that human embryonic development can proceed even if POLRMT function is substantially impaired.

In contrast, the dominant S582F model remained viable (both as hetero- and homozygotes) and appeared physiologically normal in weight gain and locomotion until 6 months of age. Nevertheless, these mice showed reduced mitochondrial transcripts, similarly to human patients carrying the S611F variant. Although *in organello* assays showed reduced de novo transcription in *Polrmt^S582F/S582F^* heart mitochondria, de novo mtDNA replication remained normal. Crucially, reduced mitochondrial transcripts were not accompanied by proteomic changes in *Polrmt^S582F/S582F^* mice. This suggests that basal mitochondrial mRNA levels exceed the minimal requirement for protein synthesis. In support of this observation, tolerance to low mitochondrial transcript levels is a phenomenon observed in other mouse models (31, 32).

While POLRMT overexpression enhances exercise performance (33), our *Polrmt^S582F/S582F^* model maintains apparently normal physiology under basal conditions despite significant molecular defects. This highlights a robust system where compromised POLRMT function remains sufficient to sustain normal physiology. However, this system is vulnerable. Our data in MEFs indicate that under metabolic stress (glucose-rich high proliferation or oxidative metabolism in galactose-containing medium), the functional threshold is exceeded, uncovering a latent phenotype. We hypothesize that in mice, the S582F mutation lowers mitochondrial transcripts to levels that are sufficient for survival under standard conditions but leaves the system vulnerable to further physiological challenges.

Interestingly, as mice aged, transcript levels tended to normalize or increase, suggesting that post-transcriptional RNA stabilizing mechanisms may counteract impaired de novo transcription (34). While the precise mechanisms triggering this stability remain to be identified, our findings suggest that the mtDNA expression machinery compensates for POLRMT dysfunction over time. Potential mechanisms could involve an upregulation of the LRPPRC/SLIRP complex, which protects mt-mRNAs from degradation, or the activation of mitochondrial protein degradation (35).

Our work establishes a framework for future gene therapy studies aimed at POLRMT-related disorders. AAV-mediated gene delivery would likely need to be administered prior to the metabolic shift at birth to be effective for severe variants. Moreover, the observation that partial restoration of mitochondrial transcripts can sustain normal physiology in *Polrmt^S582F/S582F^* mice indicates that gene therapy may not require 100% efficiency.

While the gross physiological timing and phenotypes in our models do not perfectly mirror human disease progression (for example, *Polrmt^R984C/R984C^* mice show perinatal lethality compared with the late-onset symptoms in R1013C patients), the underlying molecular phenotypes are remarkably consistent. While discrepancy in phenotypes between mice and humans can be found in mitochondrial research, the core molecular machinery of mtDNA expression is highly conserved. Our models recapitulate the mutation-specific severity gradient in transcript levels observed in patient studies: the S582F variant (corresponding to human S611F) causes intermediate transcription defects, while the R984C mutation (human R1013C), located near the active site, causes a severe loss of activity (17).

Moreover, the preservation of mtDNA copy number across our models, despite profound transcription defects, aligns with the observation that *POLRMT* patients typically do not present reduced mtDNA levels. Notably, the dominant action of the S582F mutation in mice matches the dominant behavior of the S611F mutation in human patients. The clinical report on the S611F patients was limited to general physiological descriptions such as developmental delay and hypotonia; our findings of impaired de novo transcription and reduced mitochondrial transcripts provide a plausible molecular basis for these symptoms. Ultimately, these mouse models provided unparalleled insights into the regulation of mtDNA expression and establish a robust foundation for correlating in vivo molecular defects with the diverse clinical manifestations of *POLRMT*-related disorders.

## Methods

### Sex as biological variable.

Molecular characterization of the mouse lines was performed using both sexes, although sex was not analyzed as an independent biological variable. Behavioral studies were conducted exclusively in males to minimize cohort variability. Whether these trends are fully recapitulated in females remains to be determined in future studies.

### Mouse generation and housing.

The *Polrmt*-edited mice were produced by the core facility Karolinska Center for Transgene Technologies (Karolinska Institutet, Stockholm, Sweden). Briefly, C57BL/6NCrl (Charles River Laboratories) zygotes were microinjected into the pronuclei with a pre-formed Cas9 (Merck)/sgRNA (Synthego) ribonucleoprotein complex and HDR donor oligonucleotide (Integrated DNA Technologies) in injection buffer (10 mM Tris-HCl [pH 7.5], 0.1 mM EDTA), each component at a final concentration of 20 ng/μL. The microinjected zygotes were subsequently implanted into the oviducts of pseudopregnant SOPF RjOrl:SWISS (Janvier Labs) surrogate female mice via microsurgery. Ear punch biopsies from the G_0_ offspring were lysed (36) and subjected to PCR with primers encompassing the region subjected to editing (Table 1). The PCR products were purified using the QIAquick PCR Purification Kit (QIAGEN) and subjected to restriction enzyme digestion (New England Biolabs) to reveal recombination of the HDR oligonucleotide template. The PCR amplicons from selected candidate G_0_ mice were subcloned into pMiniT 2.0 (New England Biolabs), and individual plasmid clones were subjected to Sanger sequencing (KIGene, Karolinska Institutet). Sequences were analyzed using SnapGene software.

Animals were housed in standard ventilated cages on a 12-hour light/12-hour dark cycle. They were fed ad libitum with a regular chow diet and water. Animals were sacrificed in a CO_2_ chamber and dissected immediately. Collected organs were snap-frozen in liquid nitrogen and stored at –80°C. Tissues dedicated to mitochondrial fraction isolation were kept in ice-cold PBS until homogenization.

### Behavioral testing.

The grip test was performed with a grip strength device (BioSeb). Animals were allowed to grip a metal grid with all 4 paws and then were pulled horizontally from the tail base. The maximal force was measured in grams using a digital force gauge coupled to the metal grid. The test was performed 3 times per animal, allowing them to rest between repetitions. Body weight was measured on the same day and used to normalize the results.

The rotarod test was done in a RotaRod device (Ugo Basile). The day before the test, animals were trained by being allowed to run in the rotarod at constant speed (4 rpm) for 90 seconds. This procedure was done 2 times with a 2-hour rest in between. On the test day, animals were placed in the rotarod in acceleration mode (4–40 rpm), reaching full speed after 300 seconds, and their latency to fall was recorded. Each animal was tested 5 times, and animals were allowed to rest between trials.

The open field test was done by recording of the animals for 1 hour using an ActiMot system (TSE Systems). Free horizontal activity and time spent in the center of the arena were used to investigate spontaneous locomotor activity and anxious behavior, respectively. ActiMot software was used for both data collection and analysis.

### Histology.

Mouse tissues dedicated to histopathology procedures were excised, rinsed in PBS, and embedded in OCT compound. Then they were snap-frozen in liquid nitrogen–chilled isopentane and stored at –80°C. Tissue sectioning was done using an Epredia CryoStar NX70 cryostat. Briefly, 10 μm sections were obtained, attached to poly-lysine–coated slides, and allowed to air-dry for a short time. After this procedure, slides were stored at –80°C until used. Hematoxylin and eosin staining was done using standard methods.

### COX/SDH sequential histochemistry.

COX/SDH staining was done by incubation of slides for 45 minutes at 37°C in freshly prepared COX solution (100 μM cytochrome *c*, 4 mM 3,3-diaminobenzidine tetrahydrochloride, catalase [20 μg/mL], and 100 mM phosphate buffer [pH 7.0]); slides were washed 3 times in PBS. Next, slides were incubated for 40 minutes at 37°C in freshly prepared SDH medium (130 mM sodium succinate, 200 μM phenazine methosulfate, 1 mM sodium azide, 1.5 mM nitroblue tetrazolium, and 100 mM phosphate buffer [pH 7.0]). Slides were washed 3 times in PBS, dehydrated with increasing ethanol concentrations (70%, 95%, 99%), and mounted for bright-field microscopy.

### RNA extraction and gene expression analysis.

Tissues dedicated to RNA extraction were snap-frozen in liquid nitrogen after dissection. RNA was extracted using TRIzol Reagent (Invitrogen) following the manufacturer’s guidelines, and total RNA was quantified using a NanoDrop spectrophotometer (Thermo Fisher Scientific). One microgram of total RNA was reverse-transcribed to cDNA using the High-Capacity cDNA Reverse Transcription Kit (Applied Biosystems), and 10 ng of cDNA was used in each qPCR reaction. qPCR reactions were done with TaqMan Universal Master Mix (Applied Biosystems), and gene-specific probes were also purchased from Applied Biosystems (TaqMan gene expression assays). Reactions were carried out in technical triplicates, and a QuantStudio 6 Flex (Applied Biosystems) was used for data collection. Acquired data were analyzed with QuantStudio Real-Time PCR software (v1.1). Relative RNA levels were calculated based on Ct values, and *Actb* was used as a housekeeping gene. Transcript levels were plotted relative to the wild-type group. All the probes used in this study are listed in Table 2.

### DNA extraction and relative mtDNA quantification.

DNA was extracted from mouse tissues using a DNeasy Blood & Tissue kit (QIAGEN) following the manufacturer’s instructions, and the DNA concentration was measured in a NanoDrop device.

Relative mtDNA levels were determined by qPCR, and 5 ng of total DNA was used in each reaction. Probes for *Nd1* and *Nd6* were used to detect mtDNA, and *Actb* was used to detect nuclear DNA. Relative mtDNA levels were calculated as the mtDNA/nuclear DNA ratio based on the obtained Ct values.

### Isolation of mitochondrial fractions.

Crude mitochondrial fractions were obtained by differential centrifugation procedures. Briefly, mouse tissues were first cut and washed with ice-cold PBS. Homogenization was done using a Teflon pestle and mitochondrial isolation buffer containing 320 mM sucrose, 1 mM EDTA, 10 mM Tris-HCl (pH 7.4) and 1x cOmplete protease inhibitor cocktail (Roche). The tissue homogenate was first centrifuged at 1,000*g* for 10 minutes at 4°C in a swing-out rotor. Supernatants were then centrifuged at 10,000*g* for 10 minutes at 4°C in a fixed-angle rotor. The pelleted mitochondrial fractions were then resuspended in an appropriate volume of buffer, snap-frozen in liquid nitrogen, and stored at –80°C until used.

### SDS-PAGE.

Protein content of samples was measured using a Pierce BCA Protein Assay Kit (Thermo Fisher Scientific). Twenty micrograms of total protein was loaded on precast Bolt Bis-Tris Plus Gels (Thermo Fisher Scientific) and run according to the manufacturer’s instructions. Gels were blotted onto PVDF membranes using an iBlot system (Thermo Fisher Scientific) and probed with antibodies of interest. Secondary antibodies coupled to HRP were used for signal detection together with enhanced chemiluminescence substrate (Bio-Rad). Membrane images were obtained in a ChemiDoc XRS+ (Bio-Rad), and signal intensity analysis was done using Image Lab software (Bio-Rad). Antibodies used in this study are listed in Table 3.

### OXPHOS enzyme activities.

Mitochondria-enriched fractions were used for OXPHOS enzymatic activity assays. The protein concentration was determined with a Pierce BCA Protein Assay Kit (Thermo Fisher Scientific). Mitochondria were pelleted and resuspended in a buffer containing 250 mM sucrose, 15 mM magnesium acetate, 2 mM EDTA, 0.5 g/L BSA, and 15 mM potassium dihydrogen phosphate (pH 7.2) to a final protein concentration of 1 mg/mL. Biochemical activities for mitochondrial complexes I, I+III, II, II+III, and IV were measured in an Indiko Clinical Chemistry Analyzer (Thermo Fisher Scientific) using standard methods as previously described (37). Results were normalized to citrate synthase activity.

### Sample preparation for mass spectrometry.

Mouse heart and liver tissues were thawed on ice and cut into small pieces, then transferred to a prefilled tube containing 400 μm LoBind silica beads (OPS Diagnostics) and supplemented with 300 μL of 8 M urea in 100 mM ammonium bicarbonate (Ambic) and 100× Halt protease inhibitor. The samples were sonicated in water bath for 5 minutes and frozen for a short time before being homogenized using Disruptor Genie (model SI-DD58, Scientific Industries, Inc.) at maximal speed on 2,700 rpm for 2 minutes and incubated on ice for 2 minutes; these steps were repeated 3 times. The samples were then centrifuged at 10,000*g* for 10 minutes at 4°C. The supernatant was collected, and 200 μL of 50 mM Ambic was added before protein concentration was determined by Qubit broad range assay (Thermo Fisher Scientific).

An aliquot of 5 μg samples was reduced with 2.5 μL of 100 mM dithiothreitol at 37°C for 45 minutes, alkylated with 7.5 μL of 100 mM iodoacetamide at room temperature for 30 minutes in the dark, and finally digested with addition of 0.1 μg of sequencing-grade modified trypsin (Promega) and incubated for 16 hours at 37°C. The digestion was stopped with 3 μL formic acid. The samples were cleaned on a C18 StageTip (Thermo Fisher Scientific) and dried using a vacuum concentrator (Eppendorf).

### Liquid chromatography/tandem mass spectrometry data acquisition.

Peptides were reconstituted in solvent A and 200 ng samples loaded on Evotip Pure (Evosep Biosystems) tips following the manufacturer’s sample loading protocol. Peptide separation was performed on an Evosep ENO system (Evosep Biosystems) using the standardized 30 samples per day (SPD) method, which provides a 44-minute gradient optimized for comprehensive proteome coverage. Peptides were separated on a PepSep column (15 cm length × 150 μm inner diameter) packed with 1.9 μm C18 particles (Bruker). The column was operated at room temperature without additional heating. The 30 SPD method uses a pre-formed gradient approach by which peptides are eluted from the Evotip with increasing concentrations of acetonitrile, then diluted and transferred to a storage loop before final separation on the analytical column.

The Evosep ENO system was coupled online to a timsTOF HT mass spectrometer (Bruker Daltonics) equipped with a fourth-generation TIMS-XR analyzer cartridge. Data acquisition was performed in data-independent acquisition parallel accumulation–serial fragmentation (dia-PASEF) mode, which combines trapped ion mobility spectrometry (TIMS) with data-independent acquisition. The MS1 survey spectra covered a mass range of 100–1,700 *m*/*z*. For MS2 fragmentation, 12 isocentric isolation windows were defined spanning 390–1,250 *m*/*z* in the mass dimension and 0.84–1.31 1/*K*_0_ in the ion mobility dimension. The dia-PASEF acquisition scheme was optimized to balance precursor coverage, selectivity, and sensitivity for the 44-minute gradient, with sufficient data points acquired across each chromatographic peak for accurate quantification. The TIMS-XR analyzer provided enhanced ion capacity (5-fold increase over previous generation) and improved dynamic range through its doubled ion storage volume, quadrupolar exit design, and optimized gas velocity profile. These features enabled better handling of complex samples and higher peptide loading masses without ion suppression or detector saturation.

### Data processing and analysis.

Raw data files (.d format) were processed using DIA-NN software (38) (version 2.2.0) for peptide and protein identification and quantification. Searches were performed against a theoretically generated spectral library using the following parameters: trypsin/P as the protease with a maximum of one missed cleavage allowed; carbamidomethylation of cysteine residues as a fixed modification; oxidation of methionine and N-terminal acetylation as variable modifications. A false discovery rate (FDR) threshold of 1% was applied at the peptide precursor level. MS1 and MS2 mass tolerances were automatically determined by DIA-NN’s optimization algorithms. The software utilized neural networks and interference correction to enable deep proteome coverage while maintaining high-confidence identifications. All other search parameters were maintained at DIA-NN default settings. Differential protein abundance was analyzed in RStudio (v2024.04.2+764) using the limma package. *P* values were adjusted for multiple testing using the Benjamini-Hochberg method (FDR). Proteins with FDR-adjusted *P* value less than 0.05 were considered significant. Principal component analysis (PCA) was performed using the PCAtools package, heatmaps were generated with pheatmap, and volcano plots were created in Instant Clue (v0.10.10, University of Cologne, Cologne, Germany) (39). Mitochondrial proteins were annotated using MitoCarta3.0 (7).

### In organello transcription.

Freshly isolated mitochondria from mouse heart (0.5 mg) were washed twice with ice-cold incubation buffer (25 mM sucrose, 75 mM sorbitol, 10 mM K_2_HPO_4_, 100 mM KCl, 0.05 mM EDTA, 5 mM MgCl_2_, 10 mM glutamate, 2.5 mM malate, 1 mg/mL BSA, 1 mM ADP, and 10 mM Tris-HCl [pH 7.2]). Thereafter, mitochondria were resuspended in 1 mL of prewarmed (37°C) incubation buffer supplemented with 60 μCi of [^32^P]α-UTP and incubated for 1.5 hours at 37°C in a rotating wheel. Following incubation, mitochondria were pelleted and resuspended in prewarmed incubation buffer supplemented with 2 mM non-radioactive UTP and incubated for another 10 minutes at 37°C. Finally, samples were washed 3 times in ice-cold washing buffer (10% glycerol, 0.15 mM MgCl_2_, and 10 mM Tris-HCl [pH 6.8]), and RNA was extracted using TRIzol Reagent following the manufacturer’s instructions. Samples were then separated in 1% MOPS/formaldehyde-agarose gel and transferred to Hybond-N+ membranes (GE Healthcare), and the signal was visualized by autoradiography using PhosphorImager screens (GE Healthcare) and a Typhoon 7000 FLA (GE Healthcare). For the input control, aliquots collected before RNA isolation were separated by SDS-PAGE, transferred to PVDF membranes, and blotted for voltage-dependent anion channel (VDAC). Signal quantification was performed using Image Lab software (Bio-Rad).

### In organello replication.

Freshly isolated mitochondria from mouse hearts (0.5 mg) were washed twice with ice-cold incubation buffer (see *In organello transcription*). Mitochondria were then resuspended in prewarmed (37°C) incubation buffer containing 50 μM dGTP, 50 μM dCTP, 50 μM dTTP, and 20 μCi [^32^P]α-dATP and incubated for 2 hours at 37°C. After incubation, mitochondria were washed 3 times with ice-cold washing buffer. DNA was isolated using a Gentra Puregene Tissue kit (QIAGEN) following the manufacturer’s instructions. Isolated DNA was thereafter separated in 0.8% agarose gels and transferred to Hybond-N+ membranes. The signal from the membrane was visualized by autoradiography using PhosphorImager screens on a Typhoon 7000 FLA. An aliquot of the labeled mitochondria was taken before DNA isolation, and proteins were separated by SDS-PAGE and transferred to PVDF membranes. Western blot against VDAC was used as loading control.

### Cell culture and proliferation analysis.

Primary mouse embryonic fibroblasts (MEFs) were isolated from E13.5 embryos of wild-type (+/+), homozygous *Polrmt^S582F/S582F^*, and homozygous *Polrmt^R984C/R984C^* genotypes. Cells were maintained in Dulbecco’s modified Eagle medium (DMEM) supplemented with 10% fetal bovine serum (FBS) and 1% penicillin-streptomycin at 37°C in a humidified atmosphere with 5% CO_2_.

For proliferation assays, MEFs were seeded in 96-well plates at a density of 2 × 10^3^ cells per well. To evaluate cellular sensitivity to increased metabolic demand, MEFs were subjected to a metabolic shift by replacement of standard growth medium with glucose-free DMEM supplemented with 15 mM galactose, 50 μg/mL uridine, 10% FBS, and 1% penicillin-streptomycin. Under these conditions, cells are forced to rely primarily on OXPHOS for ATP production. For pharmacological inhibition experiments, *Polrmt^S582F/S582F^* and wild-type MEFs were treated with the indicated concentrations of IMT1B (a POLRMT-specific inhibitor) or an equivalent volume of DMSO as a vehicle control.

### Cell viability and proliferation assays.

Cell viability and proliferation were monitored daily over a 5-day period using the Cell Counting Kit-8 (CCK-8) (Abcam/Sigma-Aldrich). At each 24-hour interval, culture medium was replaced with fresh culture medium containing CCK-8 reagent (10 μL per 100 μL of medium) and incubated for 1 hour at 37°C. The absorbance, serving as a proxy for viable cell number, was measured at 450 nm using a microplate reader, and background absorbance from cell-free medium was subtracted from all readings.

### Statistics.

GraphPad Prism 10 software was used to statistically analyze the data and generate the corresponding graphs. Data are represented as means ± standard error of the mean (SEM) or as otherwise stated in the figure legends. Mann-Whitney test, 2-tailed Student’s *t* test, and 1- or 2-way ANOVA were used throughout the study. The statistical test used and the number of biological replicates are stated in the figure legends.

### Study approval.

All the animal studies were approved by Regional Research Animal Ethics Committee in Stockholm, Sweden (Stockholms djurförsöksetiska nämnd) and performed in compliance with national and European law.

### Data availability.

Values for all data points in graphs are reported in the Supporting Data Values file. The mass spectrometry proteomics data were deposited to the ProteomeXchange Consortium via the PRIDE (40) partner repository with the dataset identifier PXD074595.

## Author contributions

DA, DRG, and NGL designed the studies. DA, DRG, KB, RGV, AV, RF, FAR, and CK performed the experiments and acquired and analyzed the data. DA, DRG, and NGL wrote the manuscript. All the authors participated in critical discussion and editing of the manuscript.

## Conflict of interest

NGL is a scientific founder of Pretzel Therapeutics Inc. and owns stock in this company.

## Funding support

The following organizations provided funding support:

- Swedish Research Council, grant 2025-02712 to NGL.
- Swedish Cancer Foundation, grant 243520Pj to NGL.
- Knut and Alice Wallenberg Foundation, grants 2023.0224 and 2024.0081 to NGL.
- European Research Council, Advanced Grant 101141290 to NGL.
- Novo Nordisk Foundation, grant NNF25OC0105033 to NGL.
- Swedish Brain Foundation, grant FO2025-0061-HK-269 to NGL.
- Swedish Diabetes Foundation, grant DIA2025-952 to NGL.
- Grant from the Swedish state under the agreement between the Swedish government and the county councils: grant FOUI-1001906 to NGL.
- DGAPA PAPIIT, grant IA204026 to DRG.
- Swedish Research Council, grant 2022-01477 to RF.
- Grants from Åhlén-stiftelsen, StratNeuro, and Hedlunds stiftelse to RF.
- MBB Frontiers career program at Karolinska Institutet, FAR.
- Swedish Research Council, grant 2024-02522 to FAR.
- Swedish Society for Medical Research, grant SG-24-0048-B-H-01 to FAR.

## Supplementary Material

Supplemental data

Unedited blot and gel images

Supporting data values

## Figures and Tables

**Table 1:**
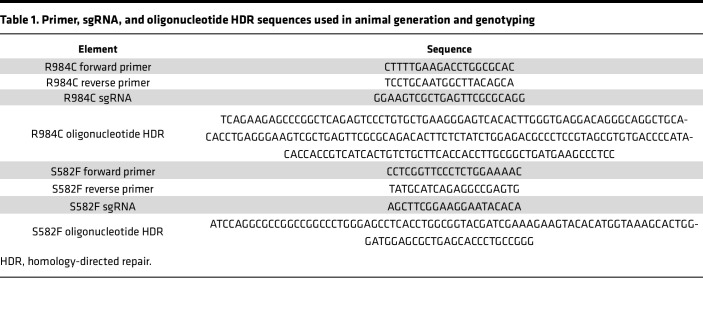
Primer, sgRNA, and oligonucleotide HDR sequences used in animal generation and genotyping

**Table 2:**
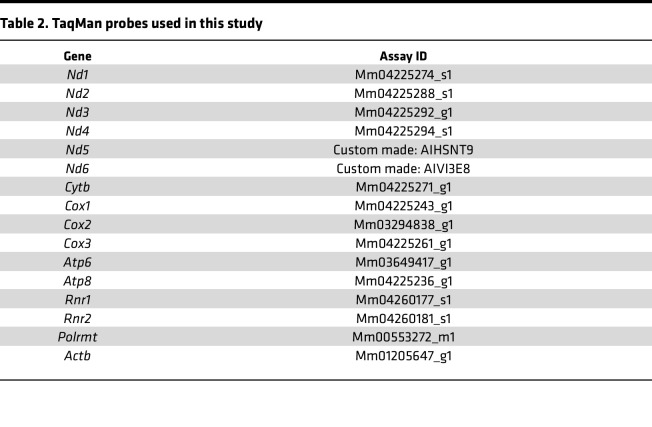
TaqMan probes used in this study

**Table 3:**
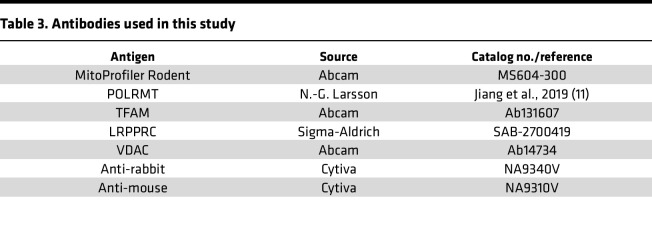
Antibodies used in this study

## Version 1 07/22/2026

Electronic publication
