# Supplementary material for: Pathogenic POLRMT variants in mice impair mtDNA transcription and affect perinatal survival
Source: JCI Insight. 2026 Jul 22;11(14):e199182. doi: 10.1172/jci.insight.199182 (PMC13461173; doi:10.1172/jci.insight.199182)
Supplement: Supplementary file 1 [file jciinsight-11-199182-s282.pdf]

Supplementary Figure 1

A

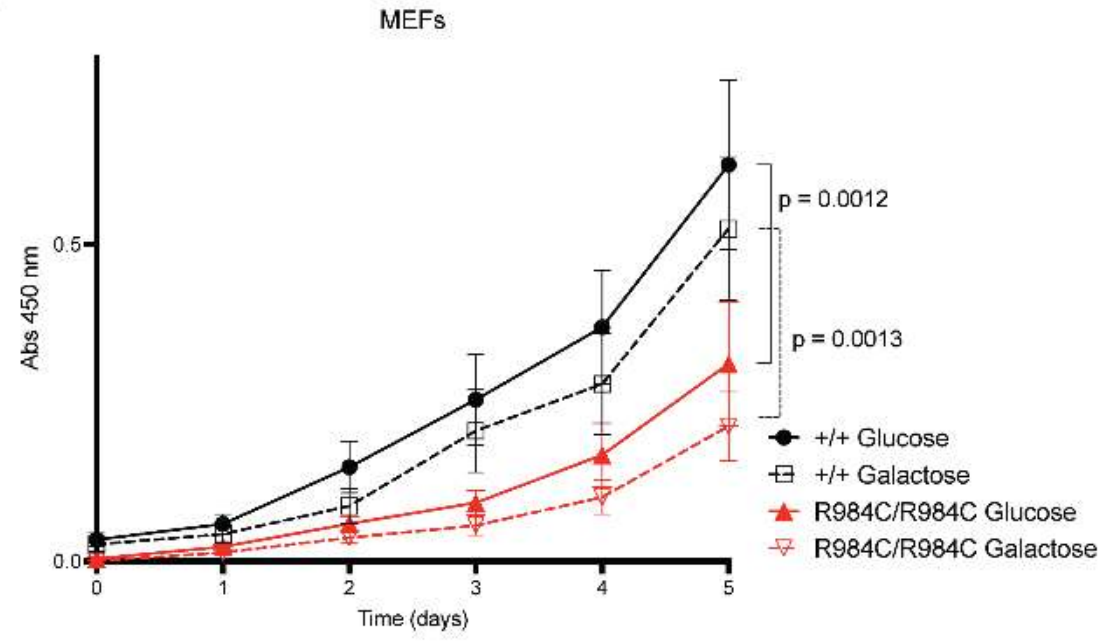

Supplementary Figure 1. Reduced proliferation in *Polrmt*$^{\mathbf{R984C/R984C}}$ MEFs.

A. Proliferation curves of wild-type (+/+) and homozygous (R984C/R984C) primary MEFs over 5 days. Cells were cultured in standard high-glucose media (solid lines) or glucose-free media supplemented with galactose (dashed lines). Cell proliferation was quantified using a CCK-8 kit. Data are presented as mean ± SEM. Statistical significance was analyzed using a two-way ANOVA with Tukey's multiple comparisons test; p-values for the day 5 time point are shown. n=3 biological replicates per condition.

Supplementary Figure 2

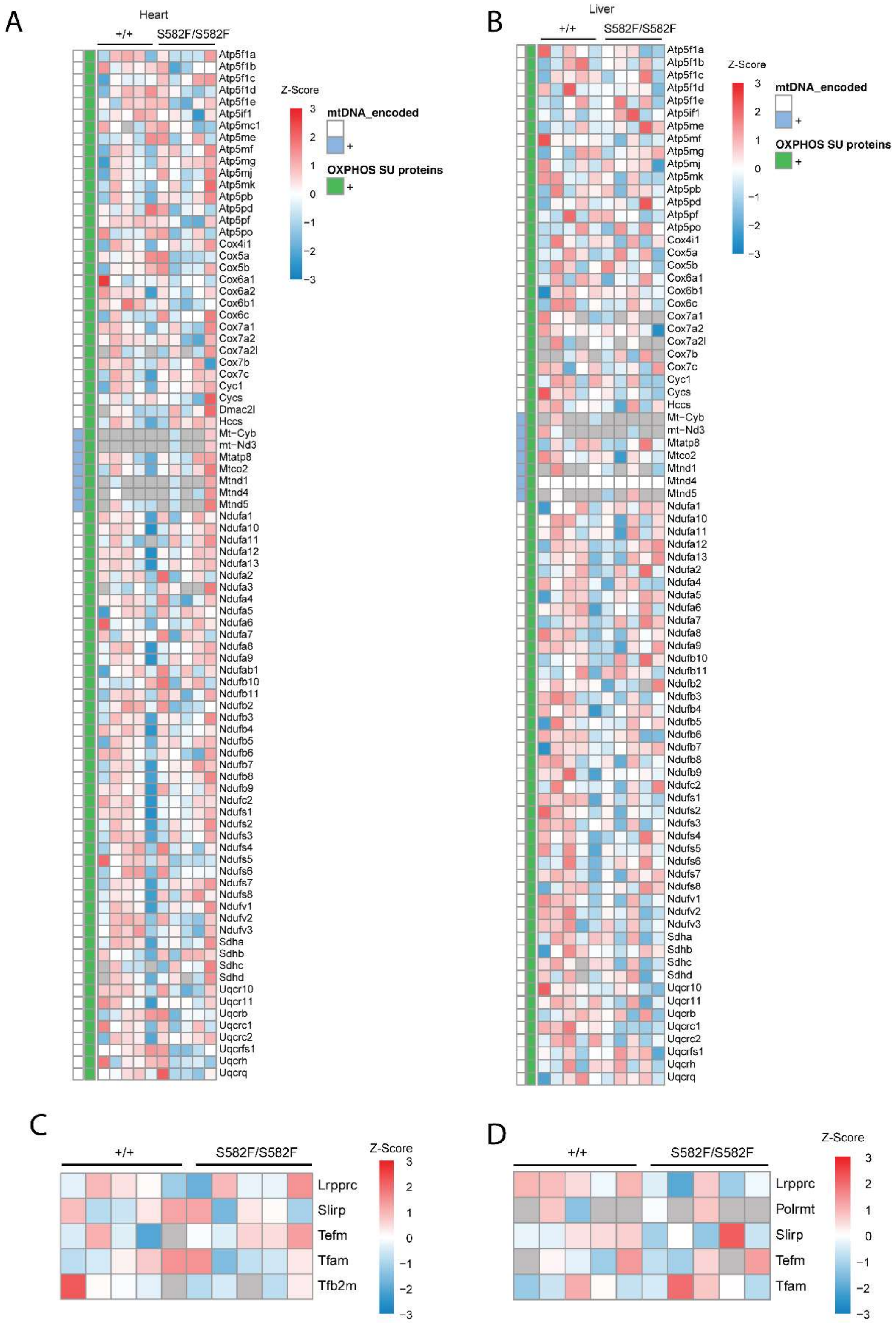

Supplementary Figure 2. Heart and liver proteomes remain stable despite the *Polrmt* S582F mutation.

A, B. Heatmaps representing the relative abundance of oxidative phosphorylation (OXPHOS) subunit proteins in **(A)** heart and **(B)** liver tissues from 15-week-old wild-type (+/+) and *Polrmt*$^{S582F/S582F}$ mutant mice. Proteins are annotated according to MitoPathways/MitoCarta 3.0. Vertical sidebars categorize proteins by functional OXPHOS complex subunits (green) and distinguish mtDNA-encoded subunits (blue). In the heatmap, color intensity represents protein abundance relative to wild-type controls, where red denotes increased abundance, blue denotes decreased abundance, and grey indicates proteins that were not detected.

C, D. Targeted analysis of key regulators of mitochondrial transcription and RNA processing (LRPPRC, SLIRP, TEFM, TFAM, TFB2M, and POLRMT) in **(C)** heart and **(D)** liver. Data are presented as Z-scores of LFQ (label-free quantification) intensities, where red indicates increased and blue indicates decreased relative abundance. No significant differences were observed between genotypes for the displayed proteins (adjusted p-value>0.05). n=5 biological replicates per genotype.

Supplementary Figure 3

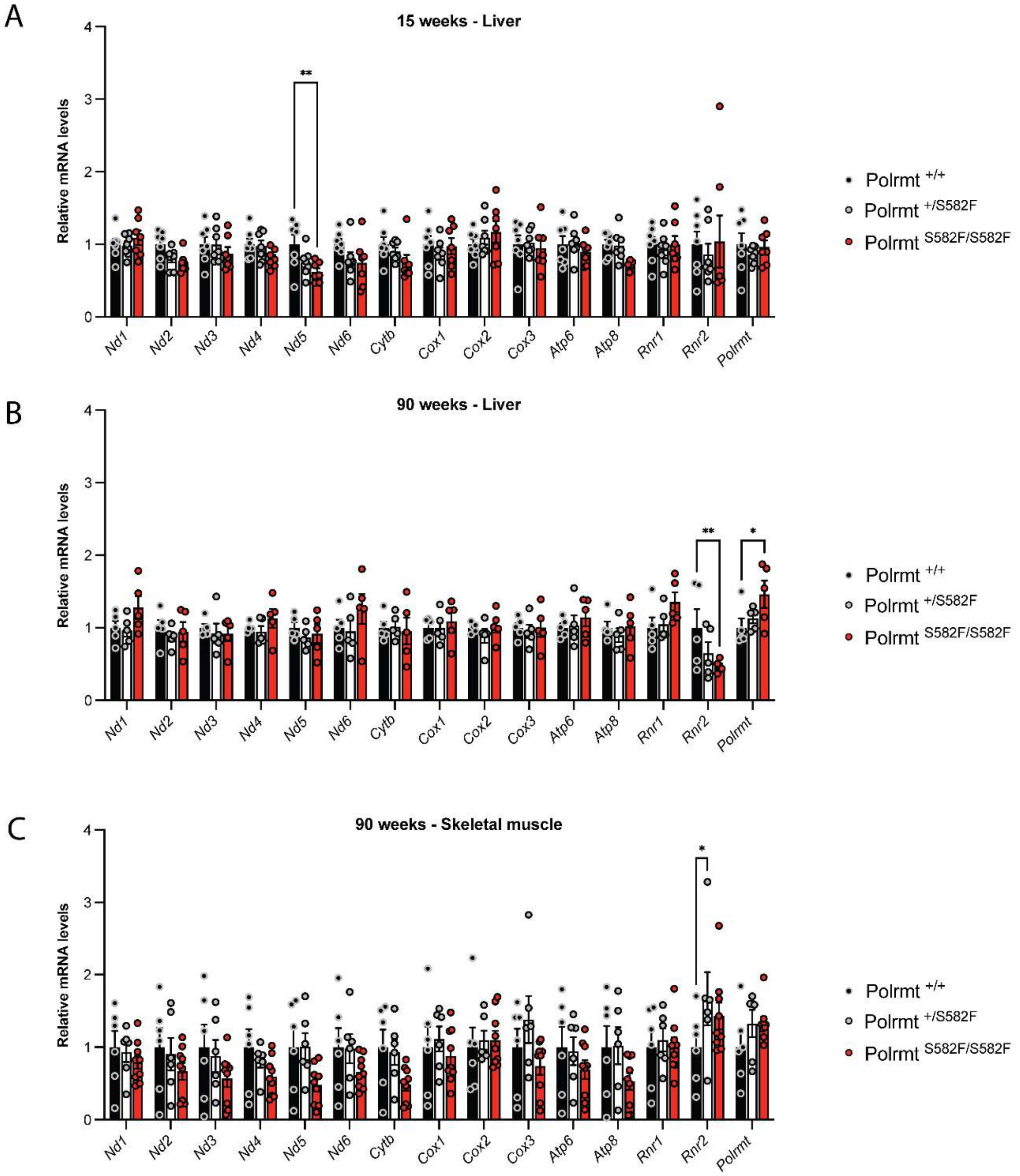

Supplementary Figure 3. Mitochondrial and *Polrmt* transcript levels in different tissues of the *Polrmt*^{S582F} mice.

A. Mitochondrial and *Polrmt* transcript levels in liver from wild-type (+/+), heterozygous (+/S582F) and homozygous (S582F/S582F) *Polrmt* knock-in mice at 15 weeks of age. Transcript levels were measured by RT-qPCR, normalized to *Actb* and plotted relative to the wild-type group. Data are presented as mean ± SEM, n=7 biological replicates per group. Data was analyzed with 2-way ANOVA followed by Dunnett's multiple comparison test, **p<0.01.

B. Mitochondrial and *Polrmt* transcript levels in liver from wild-type (+/+), heterozygous (+/S582F) and homozygous (S582F/S582F) *Polrmt* knock-in mice at 90 weeks of age. Transcript levels were measured by RT-qPCR, normalized to *Actb* and plotted relative to the wild-type group. Data are presented as mean ± SEM, n=5 biological replicates per group. Data was analyzed with 2-way ANOVA followed by Dunnett's multiple comparison test, *p<0.05, **p <0.01.

C. Mitochondrial and *Polrmt* transcript levels in skeletal muscle from wild-type (+/+), heterozygous (+/S582F) and homozygous (S582F/S582F) *Polrmt* knock-in mice at 90 weeks of age. Transcript levels were measured by RT-qPCR, normalized to *Actb* and plotted relative to the wild-type group. Data are presented as mean ± SEM, +/+ n=6, +/S582F n=6, S582F/S582F n=9 biological replicates. Data was analyzed with mixed-effect analysis followed by Dunnett's multiple comparison test, *p<0.05.

Supplementary Figure 4

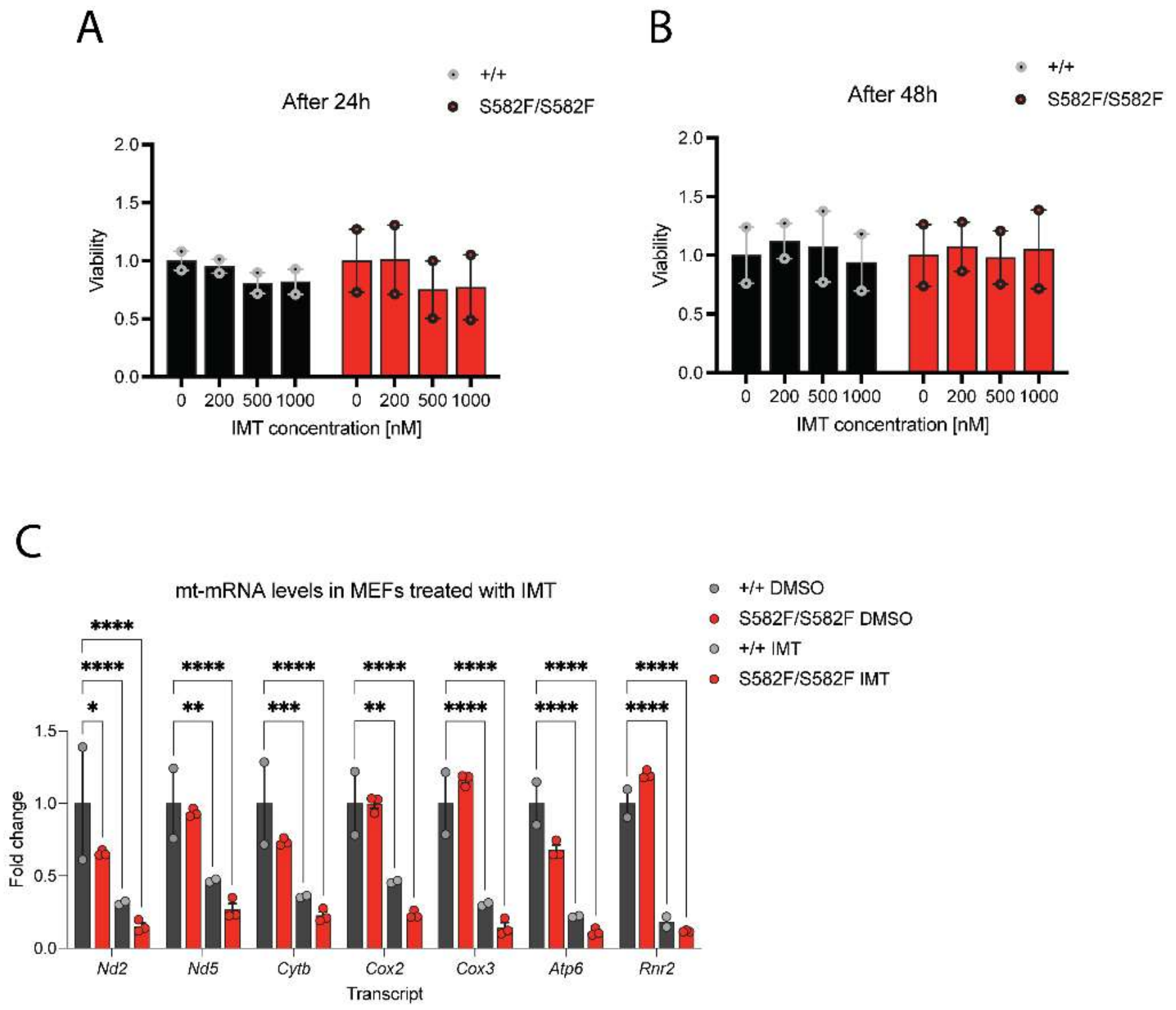

Supplementary Figure 4. Pharmacological inhibition of POLRMT reduces mitochondrial transcripts without further compromising MEF viability.

A, B. Relative viability of wild-type (+/+) and homozygous (S582F/S582F) mutant primary MEFs following treatment with increasing concentrations of the POLRMT inhibitor IMT1B (0, 200, 500, and 1000 nM) for **(A)** 24 hours and **(B)** 48 hours. Viability was normalized to the DMSO-treated (0 nM) control for each genotype. No significant dose-dependent decrease in viability was observed between genotypes at these time points.

C. RT-qPCR analysis of mtDNA-encoded transcripts in wild-type (+/+) and homozygous (S582F/S582F) mutant primary MEFs treated with 1 µM IMT1B or DMSO. Values were normalized to *Actb* and are presented as fold change relative to WT+DMSO. While IMT1B treatment robustly suppressed mitochondrial transcription in both genotypes, it did not result

in an additive growth defect. Data are presented as mean ± SEM. Statistical significance was determined by two-way ANOVA with Tukey's multiple comparisons test; ****$p<0.0001$, ***$p<0.001$, **$p<0.01$, *$p<0.05$. n=2-3 biological replicates.
