# Supplementary material for: Pathogenic POLRMT variants in mice impair mtDNA transcription and affect perinatal survival
Source: JCI Insight. 2026 Jul 22;11(14):e199182. doi: 10.1172/jci.insight.199182 (PMC13461173; doi:10.1172/jci.insight.199182)

Full unedited blots for Figure 4C

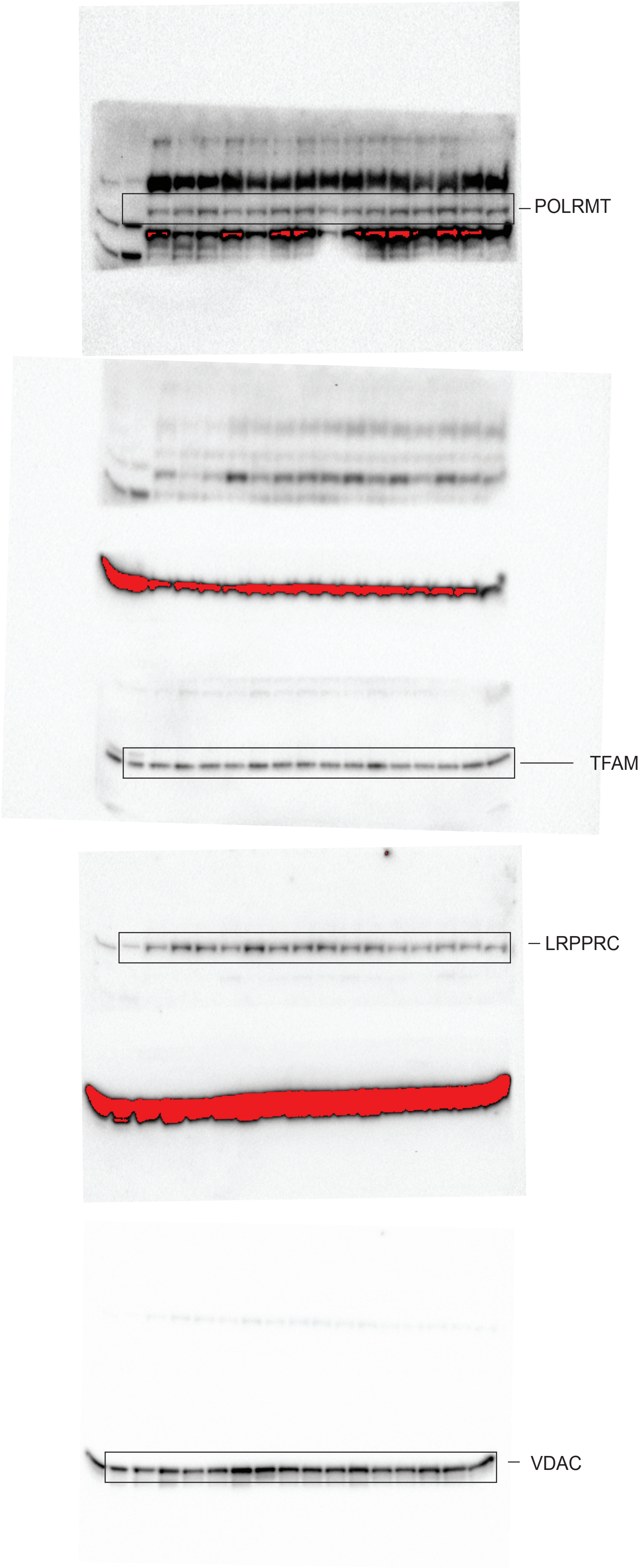

Full unedited blots for Figure 4E

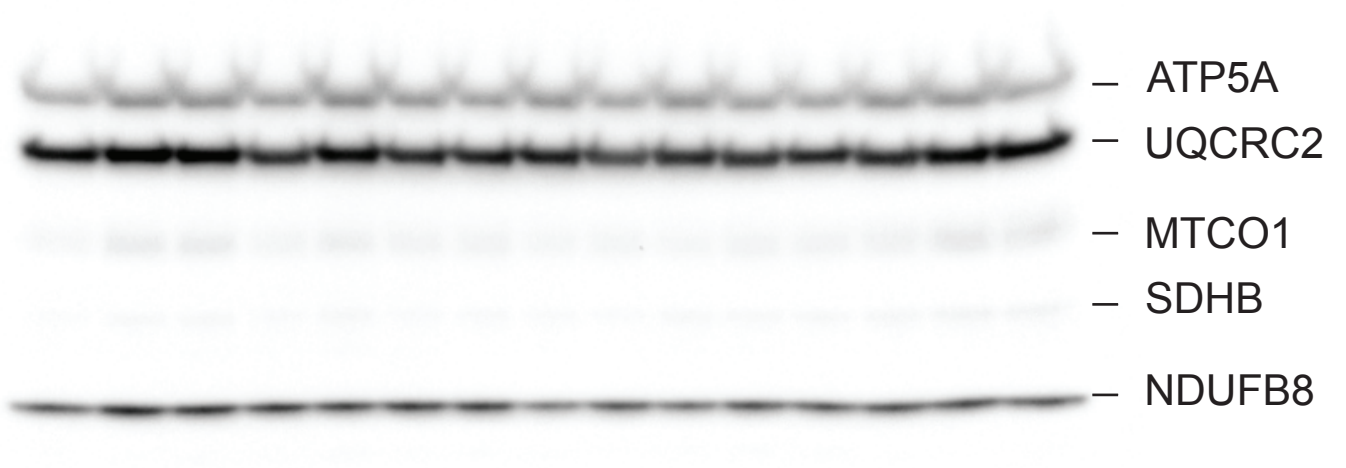

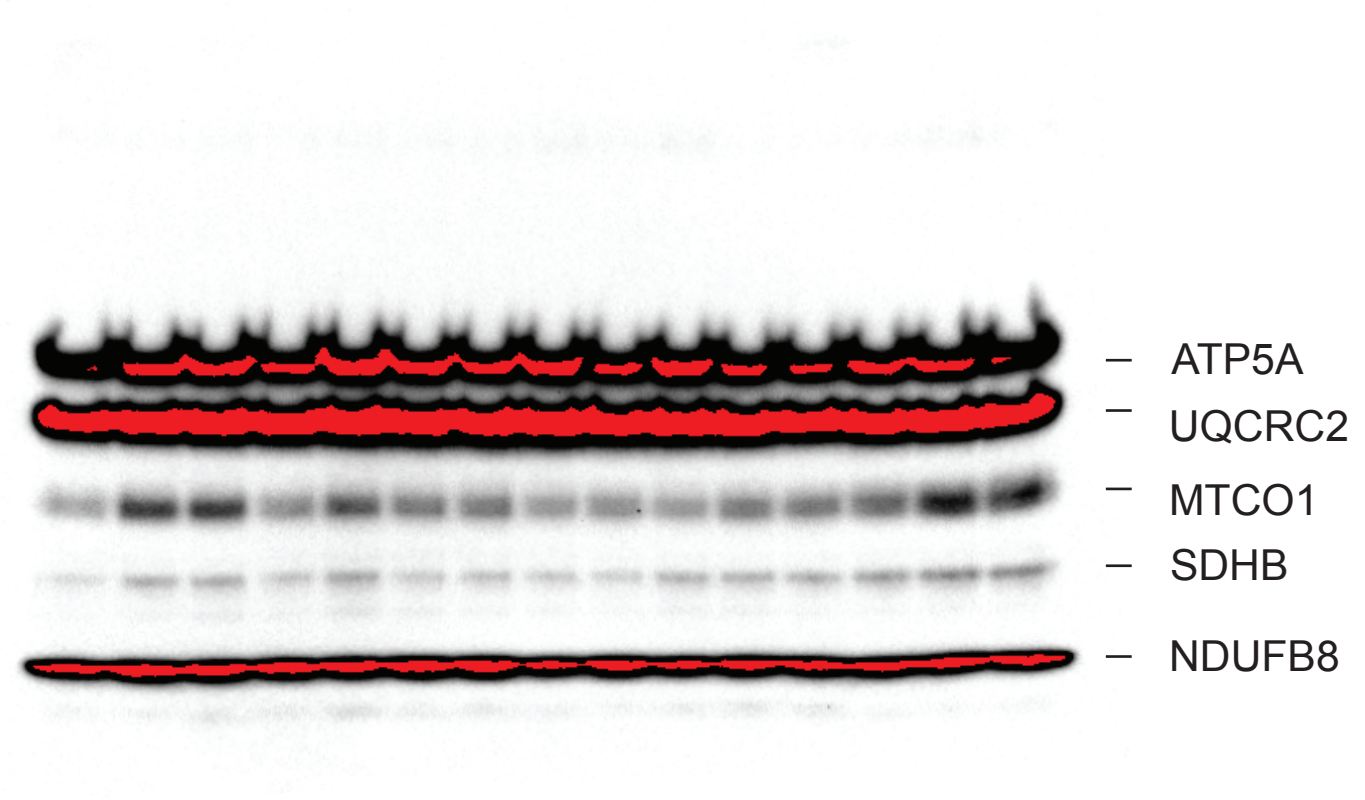

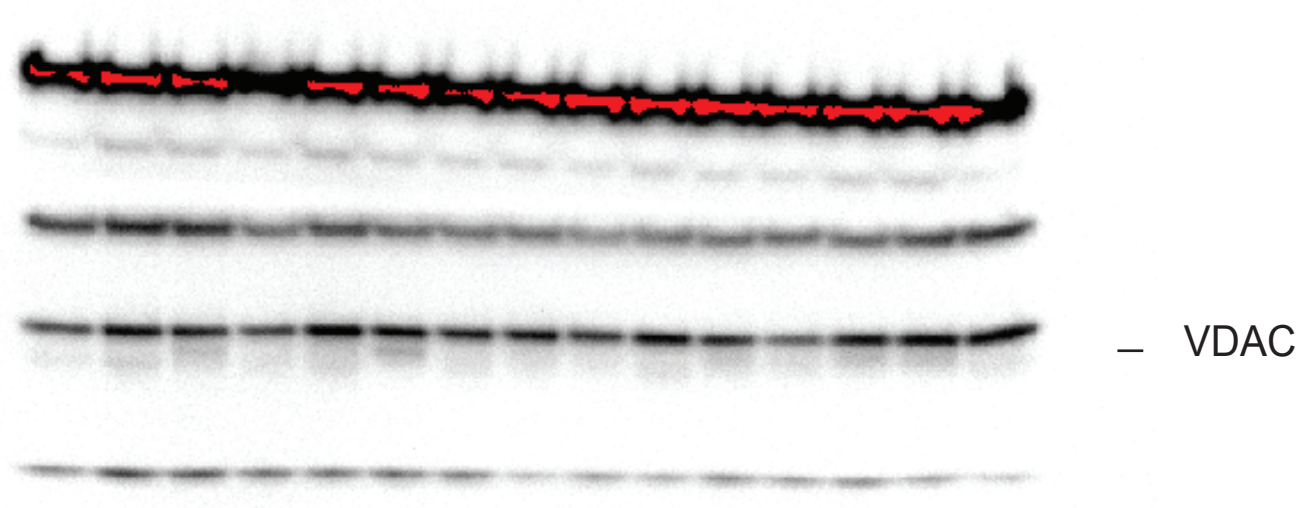

Full unedited blots for Figure 5C and 5E

VDAC,
Figure 5,
panel C

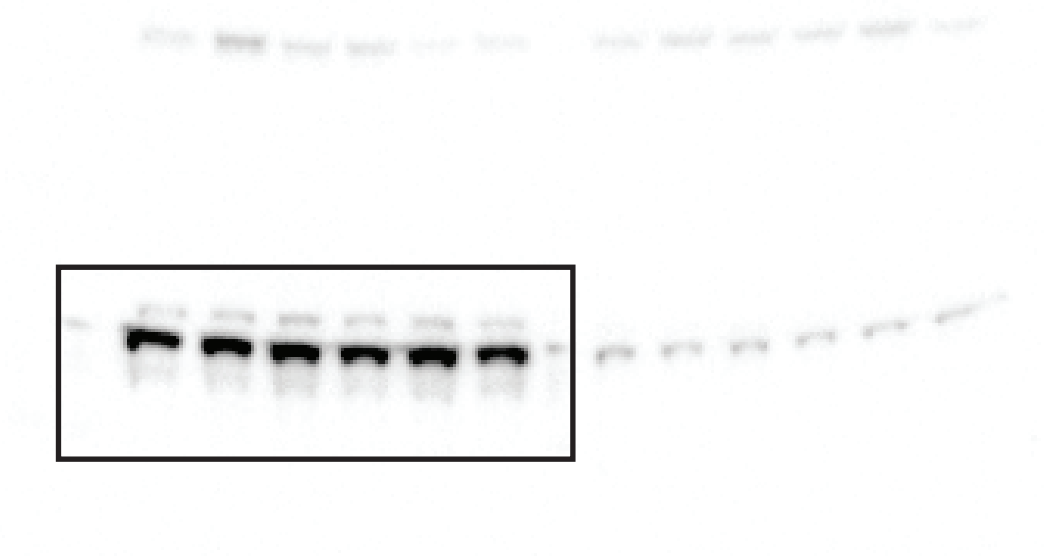

VDAC,
Figure 5,
panel E

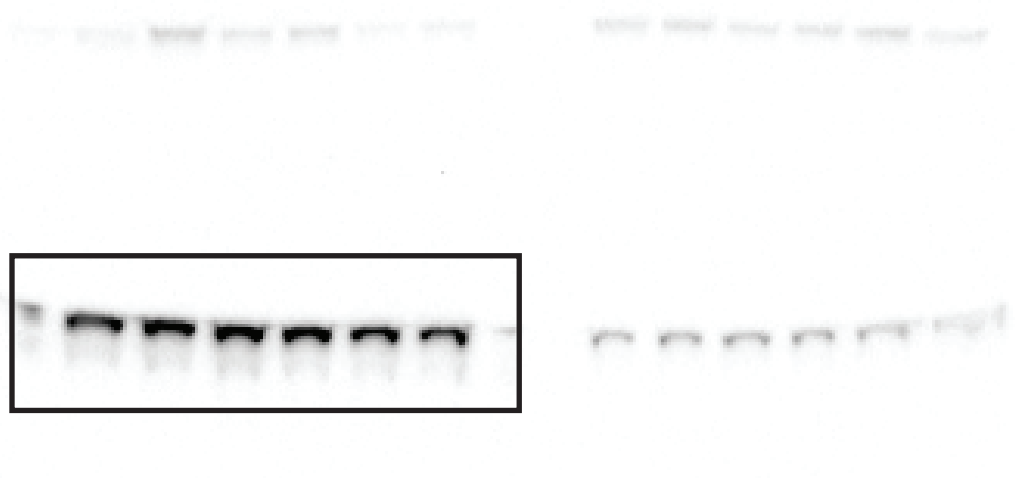

Supplement: Supplementary file 2 [file jciinsight-11-199182-s283.pdf]
